# Mannose-6-Phosphate Isomerase Functional Status Shapes a Rearrangement in the Proteome and Degradome of Mannose-Treated Melanoma Cells

**DOI:** 10.1021/acs.jproteome.4c00705

**Published:** 2024-10-18

**Authors:** Nathália de Vasconcellos Racorti, Matheus Martinelli, Silvina Odete Bustos, Murilo Salardani, Maurício Frota Camacho, Uilla Barcick, Luis Roberto Fonseca Lima, Letícia Dias Lima Jedlicka, Claudia Barbosa Ladeira de Campos, Richard Hemmi Valente, Roger Chammas, André Zelanis

**Affiliations:** † Functional Proteomics Laboratory, Federal University of São Paulo − UNIFESP, São José dos Campos, São Paulo 12231-280, Brazil; ‡ Grupo de Oncologia Experimental, Instituto do Câncer do Estado de São Paulo − ICESP, São Paulo, São Paulo 01246-000, Brazil; § Instituto de Estudos em Saúde e Biológicas, Universidade Federal do Sul e Sudeste do Pará- Unifesspa, Marabá, Pará 68507-590, Brazil; ∥ Laboratory of Biochemistry and Molecular and Cellular Biology of Fungi, Federal University of São Paulo − UNIFESP, São José dos Campos, São Paulo 12231-280, Brazil; ⊥ Laboratory of Toxinology, Center for Research, Innovation, and Surveillance in COVID-19 and Health Emergencies, FIOCRUZ, Rio de Janeiro 21040-900, Brazil; # Faculdade de Medicina da Universidade de São Paulo, São Paulo 01246-903, Brazil

**Keywords:** melanoma, phosphomannose isomerase, degradomics, metabolic rewiring, proteomics

## Abstract

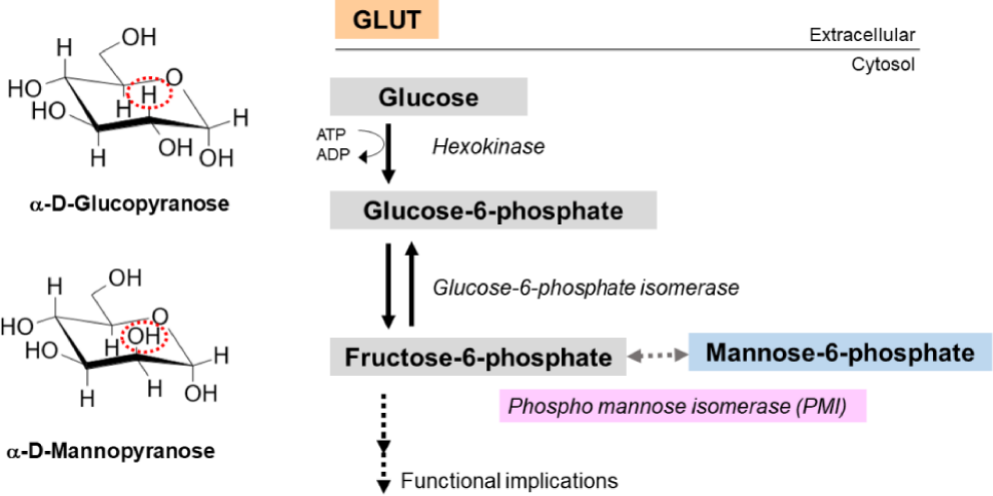

Metabolic reprogramming is a ubiquitous feature of transformed cells, comprising one of the hallmarks of cancer and enabling neoplastic cells to adapt to new environments. Accumulated evidence reports on the failure of some neoplastic cells to convert mannose-6-phosphate into fructose-6-phosphate, thereby impairing tumor growth in cells displaying low levels of mannose-6-phosphate isomerase (MPI). Thus, we performed functional analyses and profiled the proteome landscape and the repertoire of substrates of proteases (degradome) of melanoma cell lines with distinct mutational backgrounds submitted to treatment with mannose. Our results suggest a significant rearrangement in the proteome and degradome of melanoma cell lines upon mannose treatment including the activation of catabolic pathways (such as protein turnover) and differences in protein N-terminal acetylation. Even though MPI protein abundance and gene expression status are not prognostic markers, perturbation in the network caused by an exogenous monosaccharide source (i.e., mannose) significantly affected the downstream interconnected biological circuitry. Therefore, as reported in this study, the proteomic/degradomic mapping of mannose downstream effects due to the metabolic rewiring caused by the functional status of the MPI enzyme could lead to the identification of specific molecular players from affected signaling circuits in melanoma.

## Introduction

The process of tumorigenesis, often originating from cumulative somatic mutations, directly affects the cellular metabolism. A recurring pattern in tumoral phenotypes is acquiring necessary nutrients even in poor environments and utilizing them to maintain viability and increase biomass.^1^ In this context, changes in intracellular and extracellular metabolites that follow cancer-associated metabolic reprogramming profoundly affect gene and protein expression, cellular differentiation, and composition of the tumor microenvironment. Metabolic reprogramming, resulting in the deregulation of physiological cellular energy metabolism, is a ubiquitous feature of transformed cells, comprising one of the hallmarks of cancer and enabling neoplastic cells to adapt to new environments.^2^ The high energy demand imposed by altered signaling circuits, such as cell proliferation and biosynthetic processes, has significant implications for the metabolism of transformed cells. Many tumors are known to be avid for glucose, and this feature is the basis for some diagnostic methods and therapies associated with treating neoplasms. Thus, chemically modified analogs of glucose or its epimers, such as mannose, have shown promising results in oncological research, mainly associated with chemotherapy.^3−5^

Mannose is an epimer of glucose at carbon-2, and both hexoses (glucose and mannose) are internalized by the same nonspecific transporters of the GLUT family.^6^ Upon internalization, hexokinase converts mannose into its phosphorylated intermediate, mannose-6-phosphate (M6P), which may follow distinct metabolic pathways, with the most physiologically relevant being the isomerization to an intermediate of the glycolytic pathway, fructose-6-phosphate (F6P), by the action of the enzyme mannose-6-phosphate isomerase (MPI). Mannose-6-phosphate may also be directed toward the *N*-glycosylation pathway of proteins and, to a lesser extent, to produce a member of the sialic acid family, namely 2-keto-3-deoxy-d-glycero-d-galacto-nononic acid (KDN).^3,7^ Gonzalez and coworkers (2018) demonstrated, both *in vitro* and *in vivo*, that cells with low expression levels of the MPI enzyme accumulate M6P, being unable to transform it into F6P, therefore affecting its progression into the glycolytic pathway, the tricarboxylic acid cycle, and the pentose phosphate pathway. Consequently, mannose significantly impaired the growth of some tumor cells and enhanced chemotherapy.

Signaling pathways related to cell growth often require a considerable energy input, which, in turn, is reflected by the high glucose uptake in transformed cells.^1^ Increased expression of GLUT1, a glucose transporter responsible for constitutive/basal glucose uptake, is observed in melanoma.^8,9^ In this regard, the receptor tyrosine kinase-rat sarcoma virus (RTK-RAS) pathway plays a crucial role in shaping the phenotypic plasticity of neoplastic cells, including in the positive modulation of GLUT1 expression.^10^ The RTK-RAS pathway is a key circuit associated with the transduction of mitogenic signals from the cell membrane to the nucleus, the primary biological outcome of which is the regulation of cell growth and proliferation.^11−13^ Within this framework, somatic mutations in genes encoding intermediates and effectors from this pathway, such as NRAS, BRAF, and NF1, are not only drivers in melanoma but also targets for therapeutic intervention.^14,15^

In this work, we performed functional analyses and profiled the proteome landscape and the repertoire of substrates of proteases (degradome) of melanoma cell lines submitted to treatment with mannose. Our results suggest a significant rearrangement in the proteome and degradome of melanoma cell lines upon mannose treatment, whose biological implications might reflect the metabolic rewiring caused by the functional status of the mannose-6-phosphate isomerase enzyme.

## Experimental Section

### Cell Lines, Culture Conditions, and Cell Lysates

The cell line A375 (CRL-1619), an epithelial cell derived from malignant melanoma from a primary source, was obtained from the American Type Culture Collection (ATCC, USA). The cell line WM1366, a 79-year-old male with stage IV superficial spreading melanoma, was obtained from Rockland, USA. Cell lines tested negative for mycoplasma contamination. All cell lines were cultured in Dulbecco’s Modified Eagle Medium (DMEM) containing 1.5 g/L sodium bicarbonate, 100 mg/L streptomycin, 25 mg/L ampicillin, 4 mM glutamine, and 10% of fetal bovine serum (FBS). In addition, the experimental conditions used in this study comprised three culture media compositions, namely: (1) DMEM with glucose (25 mM, final concentration), (2) DMEM with mannose (25 mM, final concentration), and (3) DMEM with a mixture of hexoses (glucose and mannose, 12.5 mM each). Cells were grown in a humidified incubator at 37 °C, with 5% CO_2_. The evaluation of cell growth was performed in multiwell plates (6 wells, 10 mm diameter, Sarstedt, Germany), as three independent experiments with cell lines supplemented without (control) or with 25 mM (final concentration) of the mixture of hexoses (Man + Glc, 12.5 mM each). Cells were counted in quadruplicate by using a hemocytometer. Cell lysates were obtained as described previously.^16^ Briefly, mechanical lysis was performed with a cell scrapper and 1 mL of cell lysis buffer (2% CHAPS, 150 mM NaCl in HEPES 50 mM, pH 7.5) with the addition of a protease inhibitor cocktail (SIGMA*FAST*, Sigma, USA). Lysates were incubated in an ice bath for 30 min under mild agitation. After centrifugation (14000 *g*, 10 min, 4 °C), supernatants were removed, subjected to protein quantitation using the Bradford method,^17^ and stored at −80 °C until use. Cell lysates were independently obtained from three biological replicates derived from each cell line/experimental condition.

### Mannose-6-Phosphate Isomerase Activity

The activity of the mannose-6-phosphate isomerase enzyme was assayed colorimetrically by measuring the product of the isomerization of mannose-6-phosphate (fructose-6-phosphate), according to the method described by Dische et al. (1951)^18^ with slight modifications. Briefly, 50 μg of proteins from cell lysates were diluted in reaction buffer (20 mM Tris, 0.5 mM ZnCl_2_, pH 7.5), followed by the addition of mannose-6-phosphate (222 μM, final concentration), and the reaction mixture was incubated for 10 min at 37 °C. Next, 1.5% cysteine-HCl was added (to a 0.25% final concentration), followed by the addition of 70% sulfuric acid (60% final concentration) and the addition of an ethanolic solution of carbazole (0.003% final concentration). The reaction was incubated for 1 h at room temperature, and the absorbance of the solution was measured at 560 nm. The reaction was performed by using cell lysates from three independent experiments.

### Quantitation of Lactate in the Culture Media

The amount of lactate was measured using the enzymatic lactate test, according to the manufacturer’s recommendation (Labtest; Minas Gerais, Brazil). Conditioned media from three biological replicates from both cell lines and in each experimental condition were used. Time-course variation in lactate levels in the conditioned media was evaluated by ANOVA, followed by the Tukey’s test for multiple comparisons using GraphPad Prism 9 software (GraphPad Software Inc., USA).

### Cathepsin B Activity

Cathepsin B activity was measured using chromogenic substrate Z-Arg-Arg-pNA (Sigma, USA). The cell lysates (50 μg of proteins) were diluted in 100 mM sodium phosphate and 1 mM EDTA pH 6.0, and a solution of dithiothreitol was added to a final concentration of 5 mM. The mixture was incubated for 5 min at 37 °C. The chromogenic substrate was added to a final concentration of 100 μM, and the reaction was incubated for 30 min at 37 °C. The absorbance was recorded at 405 nm, and the amount of p-nitroaniline released during the assay was calculated using the Lambert–Beer equation based on the molar extinction coefficient of p-nitroaniline at 405 nm. The reaction was performed using cell lysates from three independent experiments. Statistical analysis (*t* test) was performed using GraphPad Prism 9 software (GraphPad Software).

### Determination of Acidic Vesicular Organelles (AVOs) and Cell Death by Flow Cytometry

The evaluation of AVOs was carried out using the acidotropic vital dye acridine orange.^19^ Cells were seeded (3 × 10^4^ cells/well) in multiwell plates (P12) in DMEM (containing 10% FBS and 25 mM Glucose). After 24 h, cultures were supplemented with the distinct experimental culture media (DMEM with 25 mM Glucose, DMEM with 25 mM Mannose, or DMEM with Glucose + Mannose, 12.5 mM each). For 3 days, every 24 h, three wells from each group (biological replicates) were removed for AVO staining and analyzed by flow cytometry. Briefly, 30 mM chloroquine was added to each well, and cultures were incubated for 1 h, followed by 3 mg/mL acridine orange (ThermoFisher, USA) for 10 min at 37 °C. The wells were washed with PBS, and the cells were detached with 0.25% trypsin-EDTA solution. The cell suspension was centrifuged for 2 min at 2000 rpm to pellet the cells, followed by resuspension in 200 μL of PBS. The cell suspension was analyzed by flow cytometry (Attune NxT Flow Cytometer, ThermoFisher, USA). A positive control group was used by culturing cells in HBSS (an amino acid-free saline solution that induces starvation, thus inducing cells to form AVOs).^20^ Cells were seeded and treated as described above for cell death analysis. After incubation under the respective experimental conditions, the wells were washed with PBS, and the cells were detached with 0.25% trypsin-EDTA solution. The cell suspension was centrifuged for 2 min at 2000 rpm to pellet the cells, followed by resuspension in 500 μL of 70% ethanol for 72 h. After removing the ethanol solution, cell pellets were washed with PBS and centrifuged for 2 min at 2000 rpm, and the cells were resuspended in propidium iodide staining solution (PBS with 100 mg/mL RNase A and 50 mg/L propidium iodide, Sigma, USA), which was then incubated for 30 min in the dark. The samples were centrifuged as described above and resuspended in PBS for flow cytometry analysis.

### *In-Solution* Trypsin Digestion and Reductive Isotopic Dimethylation Labeling

The *in-solution* trypsin digestion was performed according to the protocol described by Pessotti et al (2020).^16^ Briefly, a solution of 6 M guanidine hydrochloride (GuHCl) was added to a sample of 100 μg of protein from each cell lysate sample to a final concentration of 3 M GuHCl, followed by the addition of dithiothreitol (DTT) to a final concentration of 5 mM. The mixture was incubated at 65 °C for 60 min. Iodoacetamide (IAA) was then added to a final concentration of 15 mM, and the samples were incubated in the dark for 60 min at room temperature. DTT was added to a final concentration of 15 mM to quench the excess of IAA. The samples were cleaned up by adding ice-cold acetone (8 volumes) and methanol (1 volume), followed by the incubation of samples for 3 h at −80 °C. After centrifugation at 14 000 *g* for 10 min, protein pellets were washed twice with one volume of ice-cold methanol and then resolubilized with NaOH solution (final concentration of 2.5 mM), followed by the addition of 50 mM HEPES buffer, pH 7.5, to a final volume of 100 μL. Trypsin (Proteomics grade; Sigma, USA) was added at a 1:100 ratio (enzyme/substrate), and protein samples were incubated at 37 °C for 18 h. Tryptic peptides were differentially labeled via stable-isotope dimethyl labeling, as previously described.^21^ Experiments were carried out in groups of two samples per labeling experiment; for example: for the A375 cell line, tryptic peptides from proteins derived from cells growing in glucose were labeled with light dimethylation, whereas those derived from cells growing in the mixture of hexoses (Mannose + Glucose) were labeled with heavy dimethylation. This procedure was carried out for the two time points that were used (24 and 48 h) and for the two cell lines (A375 and WM1366). The samples were analyzed using the same LC and MS settings, and after database searching, the (log2) precursor intensities of all the samples (i.e., light and heavy intensities) were normalized, as described below, to allow intra- and interexperimental comparisons. In brief, tryptic peptides were submitted to reductive dimethylation with either light or heavy formaldehyde/cyanoborohydride solutions as follows: tryptic peptides from cell lysates grown in DMEM + Glucose 25 mM (light) vs tryptic peptides from cell lysates grown in DMEM + Glucose + Mannose (12.5 mM each) (heavy). Tryptic peptides (pH 7.5) from each sample were incubated overnight at 37 °C with either light or heavy sodium cyanoborohydride (NaBH_3_CN, light, or NaBD_3_CN, heavy) to a final concentration of 200 mM followed by the addition of formaldehyde ^12^CH_2_O (light) or ^13^CD_2_O (heavy) to a final concentration of 400 mM, resulting in mass differences of +28.031300 and +36.075670 Da for the light- and heavy-labeled samples, respectively. The reaction was terminated by adding 1 M Tris (pH 6.8 to a final concentration of 200 mM) to each sample, and the mixture was incubated for 2 h at 37 °C. Samples were then combined in a 1:1 ratio. After desalting using C18 cartridges (3M Empore SPE Extraction disks, USA), peptide samples were dried in a SpeedVac and stored at −20 °C until nanoflow liquid chromatography/tandem mass spectrometry (LC–MS/MS) analysis.

### Terminal Amine Isotopic Labeling of Substrates (TAILS)

Five hundred micrograms of proteins from the cell lysates from each cell line/experimental condition were subjected to the TAILS protocol as described previously.^22^ A solution of 8 M guanidine hydrochloride (GuHCl) was added to the incubation sample to a final concentration of 4 M GuHCl, followed by dithiothreitol (DTT) to a final concentration of 5 mM. The mixture was incubated at 65 °C for 60 min. Iodoacetamide (IAA) was then added to a final concentration of 15 mM, and the samples were incubated in the dark for 60 min at room temperature. DTT was added to a final concentration of 10 mM to quench the excess of IAA. N-termini were differentially labeled via stable-isotope reductive dimethylation, as previously described^21^ with either light or heavy formaldehyde solutions as follows: N-termini from cells grown in DMEM + Glucose 25 mM (light) vs N-termini from cells grown in DMEM + Glucose + Mannose (1.5 mM each) (heavy). Samples from each experimental condition were incubated overnight at 37 °C with sodium cyanoborohydride (NaBH_3_CN) to a final concentration of 20 mM, followed by the addition of formaldehyde ^12^CH_2_O (light) or ^13^CD_2_O (heavy) to a final concentration of 40 mM. The reaction was terminated by adding 1 M Tris (pH 6.8 to a final concentration of 100 mM) to each sample, and the mixture was incubated for 3 h at 37 °C. Samples were then combined at a 1:1 ratio. The samples were cleaned up by adding ice-cold acetone (8 volumes) and methanol (1 volume), followed by the incubation of samples for 3 h at −80 °C. After centrifugation at 18 000 *g* for 10 min, the protein pellet was washed twice with one volume of ice-cold methanol and then resolubilized with 100 mM NaOH solution (final concentration of 2.5 mM), followed by the addition of HEPES buffer, pH 7.5, to a final concentration of 25 mM in 1000 μL of reaction solution. Trypsin (Promega) was added in a 1:100 ratio (enzyme/substrate), and the mixture was incubated at 37 °C for 18 h. N-terminal peptides were enriched using a dendritic polyglycerol aldehyde polymer (Flintbox, http://www.flintbox.com/public/project/1948) as described.^22^ N-termini peptides were desalted using C18 StageTips (Empore, 3M, USA).^23^ Samples were dried in a SpeedVac and stored at −20 °C for LC–MS/MS analysis.

### Liquid Chromatography Coupled to Tandem Mass Spectrometry (LC–MS/MS)

Dimethylation labeling (DML) and TAILS samples were resuspended in 70 and 15 μL of formic acid 1%, respectively. Peptide concentrations were estimated by A_280_ reading on a Nanodrop instrument and normalized accordingly. Three biological replicates (in technical triplicate runs; 2 μg/run) were analyzed for dimethylated peptide samples, while for TAILS, only two biological replicates (in technical triplicate runs; 0.8 μg/run) were assayed. Each sample was submitted to reversed-phase nanochromatography coupled to high-resolution nanoelectrospray ionization mass spectrometry. Liquid chromatography was performed using an Easy 1200 nanoLC system (ThermoFisher Scientific, USA). Samples were initially applied, at 2 μL/min of 0.1% (v/v) formic acid in water, to a 2-cm-long trap column (100 μm inner diameter) packed with ReproSil-Pur C18-AQ 120 Å 3 μm matrix (Dr. Maisch GmbH, Germany). Next, peptides were submitted to chromatographic separation on a 39-cm-long in-house fritted column (75 μm inner diameter), packed with ReproSil-Pur C18-AQ 120 Å 1.9 μm matrix (Dr. Maisch GmbH, Germany), heated at 35 °C, and coupled to a 12-cm-long 20 μm inner diameter uncoated emitter (New Objective, Littleton, MA, USA). Fractionation was performed at 200 nL/min with 0.1% (v/v) formic acid in water and 0.1% (v/v) formic acid in 80% acetonitrile in water as mobile phases A and B, respectively. Elution was carried out with a gradient from 2% to 31% B in 117 min, up to 50% B in 37.5 min, and a final concentration increased to 100% B in 4 min. The eluted peptides were introduced directly into a Q Exactive Plus Orbitrap instrument (ThermoFisher, USA). Ionization was achieved by applying 1.9 kV to the source, setting the capillary temperature to 250 °C and alternate current (radiofrequency) level of the S-lenses at 60 V. The complete MS1 scans (300 to 1500 *m*/*z*) were acquired in the profile mode with one microscan at 70 000 resolution and an automatic gain control target value of 1 × 10^6^ with a maximum injection time of 100 ms. The 12 most intense precursor ions within the isolation window and offset of 2.0 and 0.5 *m*/*z* were selected for HCD (higher-energy collision dissociation) fragmentation with a normalized collision energy of 30 units. The MS2 spectra (200 to 2000 *m*/*z*) were acquired in centroid mode with one microscan at 17 500 resolution and an AGC target value of 5 × 10^4^ with a maximum injection time of 50 ms. Dynamic exclusion was set to 40 s, whereas peaks with unassigned charges or *z* = 1 were rejected.

### Proteomics Data Processing and Bioinformatic Analyses

Mass spectrometric (RAW) data were analyzed with MaxQuant software^24^ (version 1.6.17.0) for shotgun proteomics dimethyl labeling data. A false discovery rate (FDR) of 1% was required for both protein and peptide-to-spectrum match identifications. Mass spectrometric data were searched against a target database restricted to the taxonomy “*Homo sapiens”* (UniProt/SwissProt; 20 431 entries). This database was also combined with the sequences of 245 common contaminants and concatenated with the reverse versions of all of the sequences. Enzyme specificity was set to trypsin, and up to two missed cleavages were allowed; cysteine carbamidomethylation was selected as fixed modification, whereas methionine oxidation, glutamine/asparagine deamidation, and protein N-terminal acetylation were chosen as variable modifications. Multiplicity was set to 2 to account for the two labeling states used (light and heavy dimethyl labeling at the peptide N-terminus and lysine side chains). Peptide identification was based on a search with an initial mass deviation of the precursor ion of 7 ppm, and the fragment mass tolerance was set to 0.02 Da, with the “requantify” and “match between runs” functions of MaxQuant software enabled. As is observed from complex proteomes such as vertebrates, peptides can be shared between homologous proteins or splice variants, leading to “protein groups”. The first protein entry was selected as representative for each protein group in the MaxQuant’s “proteinGroups.txt” file. All the light and heavy peptide intensities were log2-transformed and quantile-normalized using the “preprocessCore” library in R scripting and statistical environment to correct for intraexperimental variation.^25,26^ Statistical analysis (*t* test) was performed in R, and proteins with an adjusted *p*-value < 0.05 and log_2_(fold change) > 1 and < −1 were considered differentially abundant. The “pheatmap” library generated heatmaps of selected proteins in R. Before generating the heatmaps, the median of log2(quantile-normalized light and heavy peptide intensities) was taken. Soft clustering analysis was performed in R using the “mfuzz” library.^27^ Prior to clustering, the median of log2-transformed abundance values (light or heavy intensities) was subjected to quantile normalization in all biological replicates, and the abundance values (z-scores) were clustered (parameters: *c* = 4, *m* = 1.25). Correlations between replicates were also calculated in R by using the Pearson correlation coefficient. Identified proteins were annotated according to the information associated with their main functions, available under the gene ontology (GO) “biological process” category at the UniProt database, using the “Retrieve/ID mapping” tool (https://www.uniprot.org/id-mapping/).

Mass spectrometric (RAW) data from TAILS experiments were analyzed within the Trans-Proteomic Pipeline platform^28^ (v.6.1 Parhelion; Build 202 206 022 232–8676). Briefly, RAW files were converted to the mzXML file format and searched with the Comet search engine^29^ (version 2021.01, rev. 0) against the same UniProt/SwissProt database described above. Peptide identification was based on a search with a mass deviation of the precursor and fragment ions of 20 ppm. As primary amine dimethylation prevents trypsin cleavage at dimethylated lysine residues, enzyme specificity was set to semi-Arg-C, and at least two missed cleavages were allowed. Separate searches were carried out to account for the two cell lines/labeling states used (Glucose 25 mM and Glucose + Mannose 12.5 mM, light and heavy dimethyl-labeled peptides, respectively). Free N-terminal peptide searching was carried out by selecting the light (+28.03 Da) and heavy (+36.07 Da) dimethylation as fixed modifications at the peptide N-terminus and lysine side chains. Acetylated N-termini were searched by selecting N-terminal acetylation (+42.01 Da) and heavy (or light) lysine side chain dimethylation as fixed modifications. For all searches, cysteine carbamidomethylation was set as a fixed modification, whereas methionine oxidation and glutamine/asparagine deamidation were selected as variable modifications. Protein identification was accepted after estimating the false discovery rate calculated based on the score distributions in the output of the Comet search engine. Search results were filtered with PeptideProphet to *a* ≥ 99% confidence interval, corresponding to a false discovery rate (FDR) of less than 1%. Protein identifications were accepted if they contained at least one identified unique peptide. The peptide list and the corresponding accession numbers were submitted to positional analysis using the TopFIND knowledge base^30^ (http://clipserve.clip.ubc.ca/topfind). Mutational data from melanoma patients were retrieved from the cBioPortal for Cancer Genomics (cbioportal.org).^31^

## Results and Discussion

### Melanoma Cell Sensitivity to Mannose Correlates with Mannose-6-Phosphate Isomerase Activity

**Figure 1:**
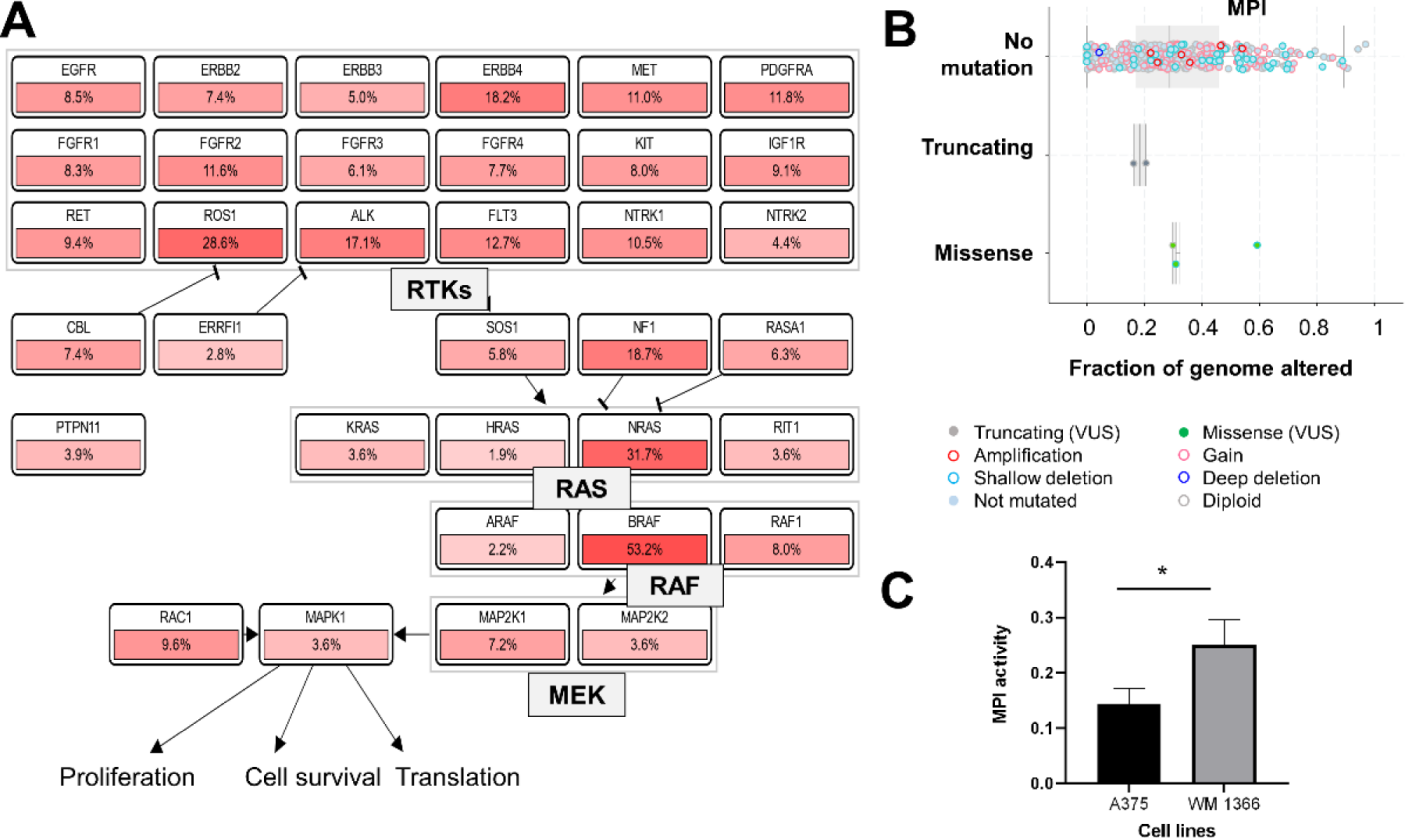
Mannose-6-phosphate isomerase (MPI) mutational status in melanoma patients and MPI enzymatic activity in cell lines used in this study (A375 and WM1366). (A) Mutational status of genes from RTK-RAS pathway and involved in GLUT1 expression (boxes contain gene names along with their respective frequency of mutation) and (B) genetic alterations in MPI. Data from 363 patient samples [The Cancer Genome Atlas – TCGA, obtained in cbioportal.org – PanCancer/skin cutaneous melanoma (VUS – Variant of Unknown Significance)]. (C) PMI activity (O.D. at 560 nm × 10) of lysates (50 μg of proteins) from cells grown in DMEM with 25 mM glucose medium for 24 h. Data (mean ± S.D.) are representative of three independent experiments (* *p* < 0.05, *t* test).

The burden of somatic mutations during tumorigenesis often affects related signaling pathways, deregulating genes encoding proteins associated with interconnected biological processes.^32^ For example, patients with melanoma frequently display somatic alterations in genes encoding proteins from the RTK-RAS signaling pathway (Figure 1A), and accumulated evidence has shown that such alterations correlate with the upregulation of genes encoding proteins related to glucose metabolism, including GLUT transporter.^33^ Thus, proteins related to key metabolic pathways may offer promising opportunities for targeted cancer therapies. In this context, we interrogated data from The Cancer Genome Atlas (TCGA, PanCancer Atlas) to investigate the mutational status of the mannose-6-phosphate isomerase (MPI) gene in cutaneous melanoma patients. While mutations in genes encoding RAS and RAF proteins are recurrent (i.e., BRAF mutation accounts for more than 50% of the observed mutations), MPI alterations were found in less than 4% of the patient samples (Figure 1B); indeed, MPI gene transcription and protein levels are low in tissues such as kidney, pancreas, and skin (Figure S1A,B) and significantly vary among skin cancer cell lines (Figure S1C). So, we reasoned that this expression pattern might be conserved even in malignant transformed melanocytes (i.e., the tumoral cell lines used in this study). Therefore, we profiled the MPI gene product’s enzymatic activity in two melanoma cell lines: A375, a melanoma cell line from a primary source, and WM1366, a cell line obtained from a patient with stage IV superficial spreading melanoma. These cell lines differ from each other mainly concerning the 1799 *T* > A mutation in the BRAF gene, leading to the V600E alteration in the gene product (B-Raf protein); such a mutation is present in the A375 cell line and absent in WM1366 (although the latter displays mutation in NRAS and CDKN2A genes). MPI activity was significantly higher in the WM1366 cell line (Figure 1C).

**Figure 2:**
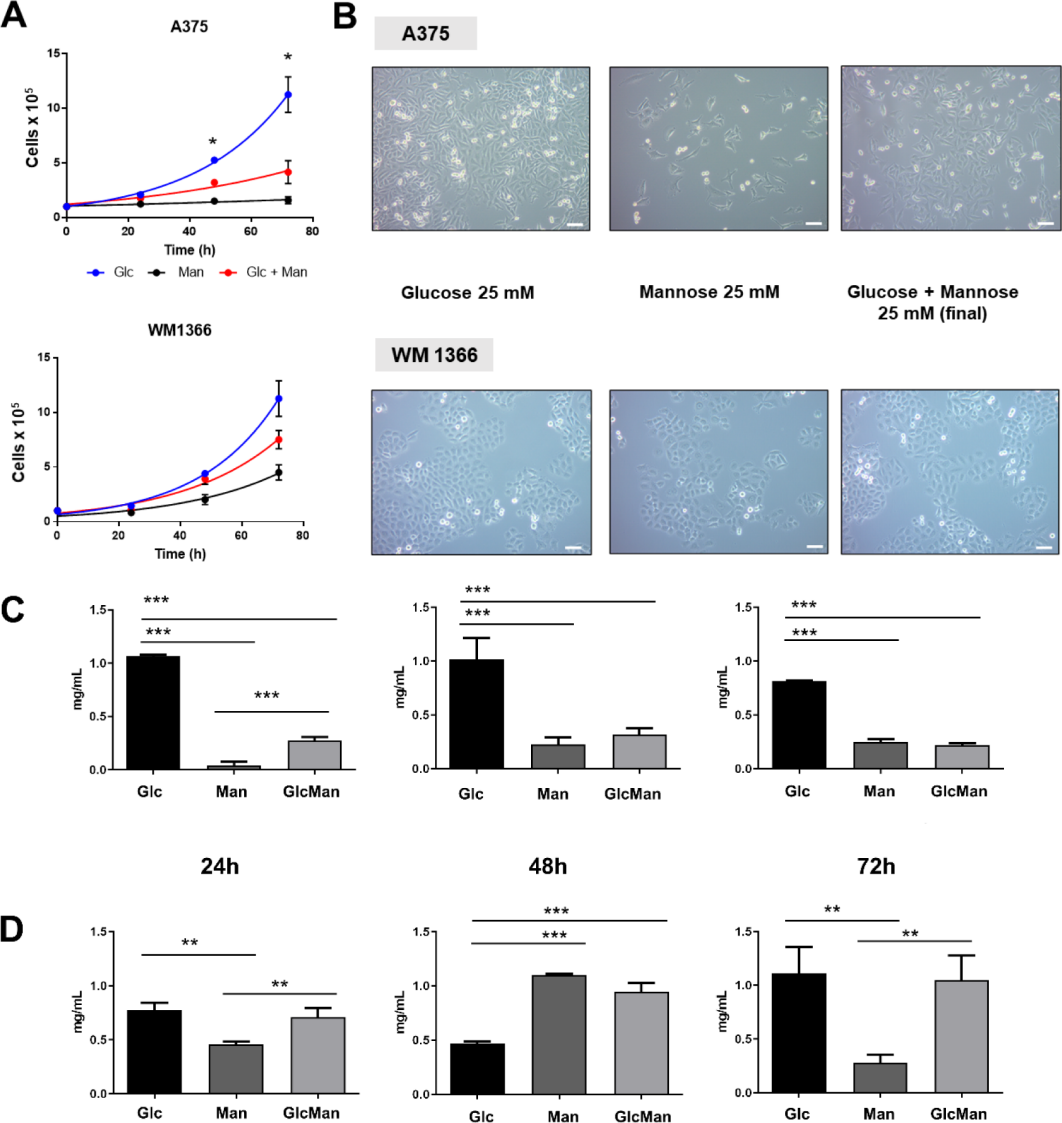
Mannose affects the growth of melanoma and the lactate levels in the conditioned media of melanoma cell lines. (A) Growth curves of A375 and WM1366 cells supplemented or not (control) with 25 mM (final concentration) of individual hexoses or their mixture (Man + Glc). Cells were counted in quadruplicate using a hemocytometer. Data (mean ± S.D.) are representative of three independent experiments and were analyzed by two-way ANOVA with Geisser–Greenhouse correction, followed by multiple comparisons correction with Tukey’s test (* *p* < 0.05). (B) Phase contrast microscope images of cell cultures after 72 h of cultivation under the aforementioned experimental conditions (scale bar = 200 μm). Time-course variation in lactate levels in the conditioned media of (C) A375 and (D) WM1366 melanoma cell lines. Data (mean ± S.D.) are representative of three independent experiments (*** *p* < 0.0001, ** *p* < 0.005; ANOVA followed by Tukey’s test for multiple comparisons).

Moreover, the sensitivity to mannose mirrored the activity of the MPI enzyme as observed in cell growth curves, with the A375 cell line being more sensitive to this hexose, mainly after 48 and 72 h of experimentation (Figure 2A,B). We observed that the experimental condition comprised by mannose alone was detrimental for the A375 cell line, with negligible growth after 72 h. The mixture of hexoses (Mannose + Glucose, 12.5 mM each) resulted in slower growth when compared to the condition with glucose alone (25 mM). Conversely, when incubated with mannose alone or in combination with glucose, the WM1366 cell line displayed exponential growth, although less pronounced when compared to the condition with glucose alone.

Unlike cells in a resting state, rapidly proliferating cells use glycolysis TCA/cycle intermediates for biosynthetic reactions and NADPH production.^1^ In such reprogramming of carbon metabolism processes, pyruvate is often reduced to lactate, which is ultimately secreted to the extracellular environment, a hallmark of the Warburg effect of concurrent fermentative and oxidative metabolism taking place at the same time. Thus, we evaluated the lactate levels in the conditioned medium (CM) of cells submitted to treatment with the hexoses. The CM from the A375 cell line displayed high lactate levels when cells were cultured in a medium with glucose as a sole source of sugar (Figure 2C), regardless of the incubation time (i.e., 24 to 72 h). In contrast, A375 cells poorly metabolized mannose or the mixture of hexoses into lactate, as observed by the low lactate levels in the respective conditioned media. On the other hand, the WM1366 cell line could efficiently metabolize either glucose, mannose, or the mixture of hexoses into lactate (Figure 2D). Although the efficiency varied over time, the ability to metabolize mannose (either alone or in combination with glucose) was superior in the WM1366 cell line, which displayed a higher MPI activity compared to the A375 cell line.

Since mannose-6-phosphate isomerase activity seemed lower in the A375 cell line, it is expected that, in a medium comprised of mannose only, the failure to fully convert M6P into F6P results in the accumulation of the former, thereby causing hexokinase inhibition and impairing the downstream glycolytic reactions, as reported previously.^3^ Such an outcome is expectedly different for a cell line displaying higher MPI activity, like the WM1366 cell line, and when the culture medium has glucose in addition to mannose. The higher activity of MPI in WM1366 may explain the fluctuations in lactate levels observed in the culture medium when cells were grown in the presence of mannose. On the other hand, the BRAF-mutant cell line, A375, could not accommodate such metabolic constraints when growing in a mannose-containing medium.

### Mannose Induces a Proteome Rearrangement in Melanoma Cell Lines, with Functional Implications

Since the culture condition composed of mannose alone was detrimental to the A375 cell line and, to a lesser extent, to the WM1366 cell line, the effect of mannose was evaluated by combining this hexose with glucose (12.5 mM of each hexose in culture medium). Using reductive isotopic dimethylation, we quantitatively evaluated the proteome of both cell lines subjected to the incubation with the mixture of hexoses (or glucose alone, as a control) for 24 and 48 h (Table S1). Overall, quantitative values for biological replicates were in good agreement, as observed by Pearson correlation coefficients ranging from 0.81 to 0.97 (Figure S2A–D).

**Figure 3:**
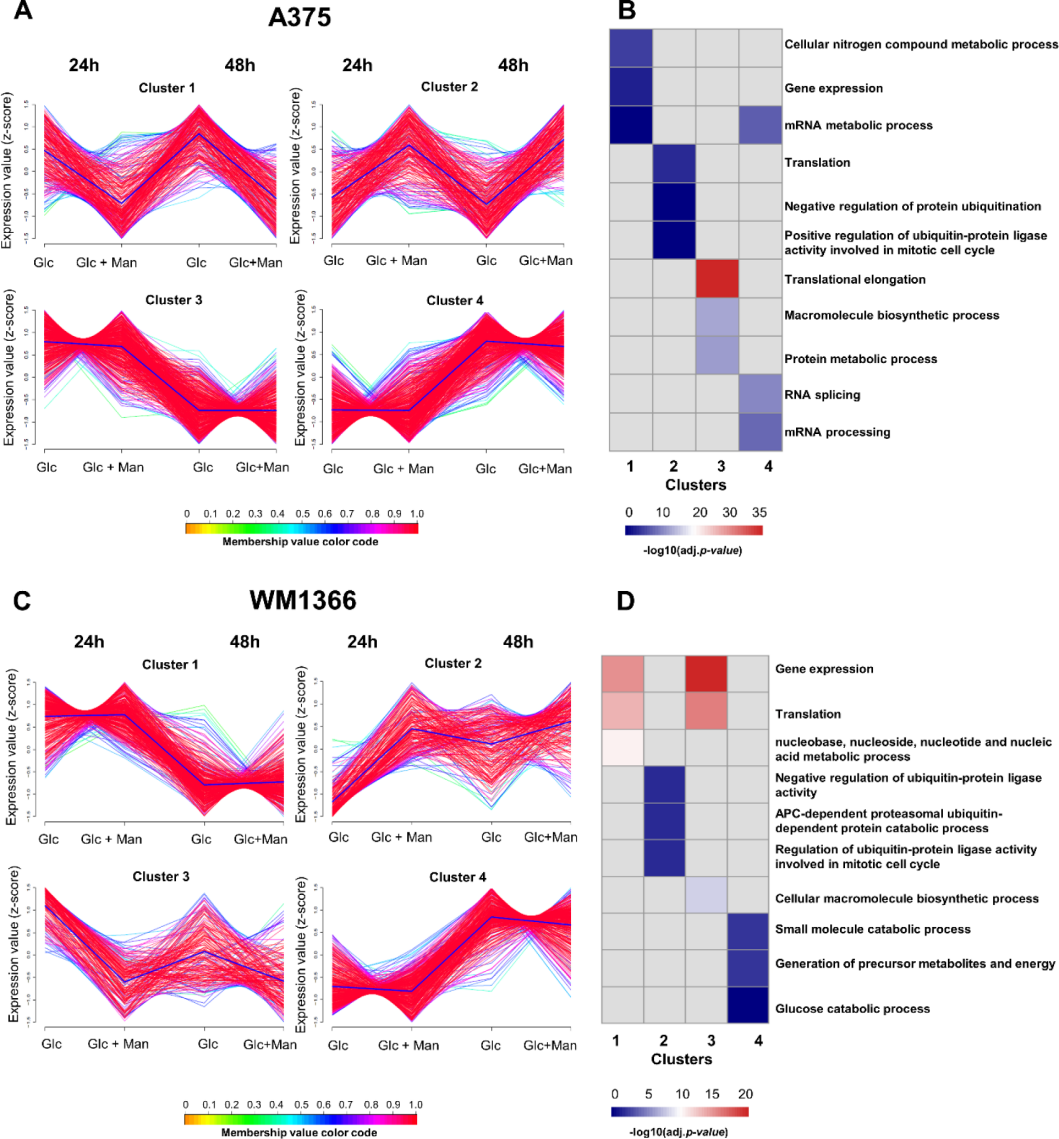
Mannose induces a proteome rearrangement in melanoma cell lines. A soft clusterization analysis (clusters generated by fuzzy c-means) of protein abundance values together with gene ontology (GO) enrichment across the different experimental conditions enabled insights into their functional implications in (A and B) A375 and (C and D) WM1366 cell lines. The *y*-axis corresponds to both cell lines’ quantile-normalized log2 median protein intensity values. Abundance values were standardized (z-score). Each abundance profile is color-coded according to its membership value for the respective cluster (membership color bar).

To obtain an initial landscape of protein quantitative values across the different experimental conditions and to enable insights into their functional implications, soft clustering analysis together with gene ontology (GO) enrichment was performed. Most clusters presented highly consistent abundance values (i.e., membership values ≥0.7), which may reveal strongly coexpressed proteins^27^ (Figure 3; Tables S2 and S3). Out of the resulting four clusters for the A375 cell line, clusters 3 and 4 displayed proteins whose quantitative values varied with assay time (24 and 48 h) experimentation, regardless of the experimental treatment (Figure 3A,B). For example, after 48 h, proteins related to biosynthetic processes (translational elongation/protein metabolism) displayed lower abundance (cluster 3), whereas those associated with mRNA metabolism (splicing/processing) displayed increased abundance (cluster 4). On the other hand, the effect of mannose on the proteome of the A375 cell line was better visualized in clusters 1 and 2, in which specific patterns of abundance were observed. Proteins related to gene expression presented lower abundance when cells were treated with a mixture of hexoses (cluster 1); in addition, those related to protein metabolism, such as negative regulators of protein turnover, displayed higher abundance upon the treatment with a mixture of hexoses (cluster 2). Similarly, out of the four clusters found for WM1366, clusters 1 and 4 presented proteins whose abundance varied according to the time of incubation; proteins related to gene expression and translation processes displayed lower values after 48 h of experiment (cluster 1), and those mainly associated with glucose catabolic processes showed higher abundance after 48 h of incubation (cluster 4) (Figure 3C,D). The effect of mannose was observed in clusters 2 and 3, with a higher abundance of proteins mainly related to protein turnover (cluster 2) and a lower abundance of proteins related to gene expression and protein biosynthesis (cluster 3).

Overall, mannose induced a rearrangement in the protein abundance profile of both melanoma cell lines, and the main biological outcomes were related to gene expression and protein turnover (synthesis/degradation). Interestingly, opposite trends were observed for related processes such as gene expression and translation (e.g., Figure 3A; clusters 1 and 2). Furthermore, proteins involved in the negative regulation of protein turnover displayed higher abundance in the presence of mannose. As a likely outcome of such a result, one might expect that the protein content of cells subjected to the administration of mannose could be affected. In fact, the protein content of both cell lines increased when cells were cultured in a medium containing mannose (Figure S3). Whether this is a direct result of the upregulation of negative regulators of protein turnover remains to be confirmed. It is worth mentioning that the results related to proteins associated with proteostasis could be due to the timescale of such biological processes; transcription and translation require a significantly distinct timescale for completion.^34^ Therefore, our proteomics analysis may be considered a snapshot of a rather complex biological process. Protein synthesis is among the main costly biological processes in metazoans,^34^ and accumulated evidence shows that metabolic rewiring is a relevant strategy for energy acquisition by tumoral cells.^1,33^

**Figure 4:**
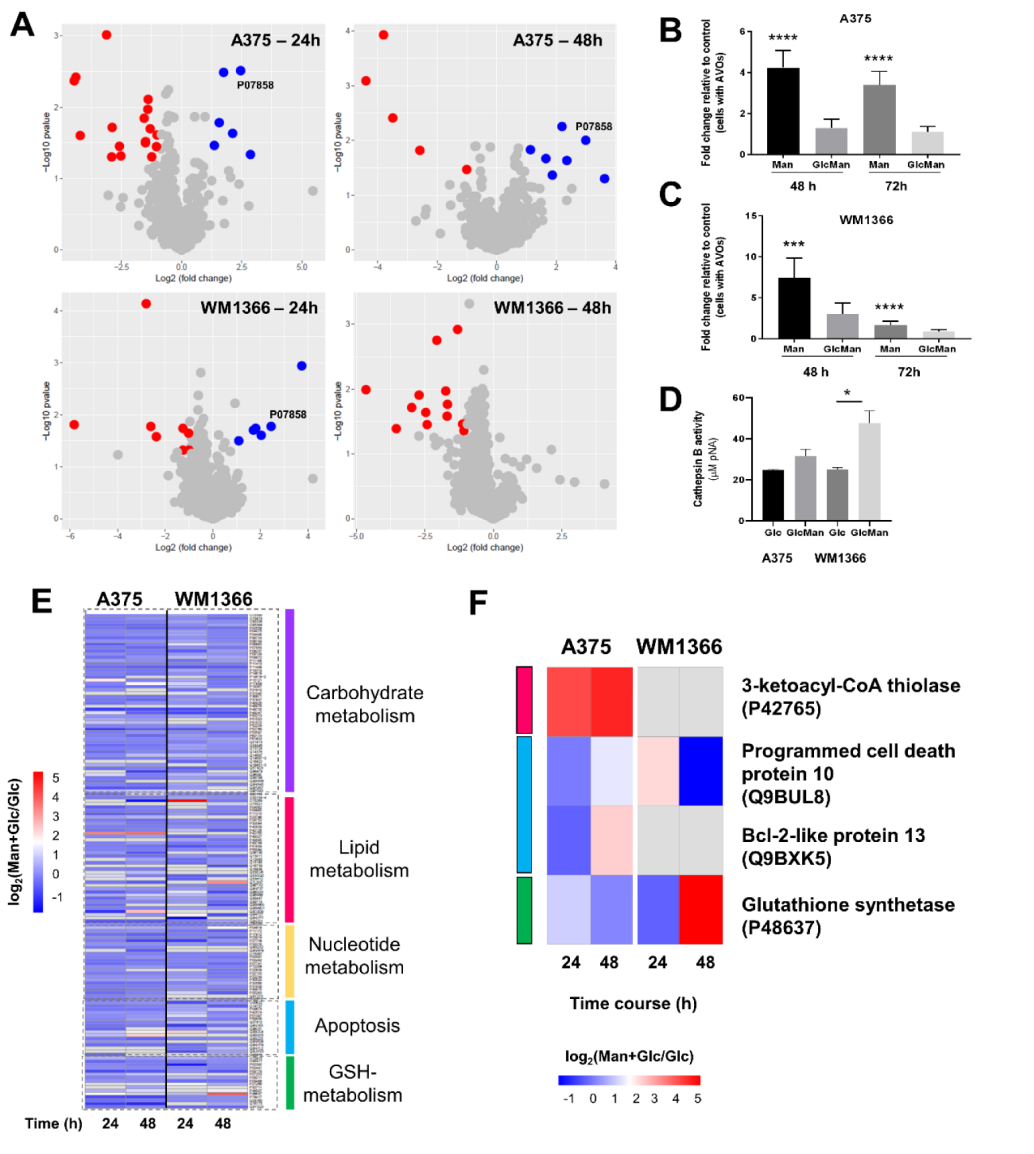
Differential protein abundance analysis upon mannose administration and its functional implications. (A) Differentially abundant proteins were identified by reductive isotope dimethylation of peptides derived from shotgun proteomics analyses of proteins from both cell lines in the two culture conditions (Glucose alone or Man + Glc) and after 24 or 48 h of incubation. Red circles denote proteins whose abundance was higher in the glucose-alone medium (glucose, 25 mM), whereas blue circles denote proteins whose abundance was higher in the culture medium containing the mixture of hexoses (Mannose and Glucose, 12.5 mM each). UniProt accession number (P07858) for the lysosomal enzyme cathepsin B is displayed. (B and C) Acidic vesicular organelle (AVO) content was evaluated by flow cytometry, using the acidotropic vital dye acridine orange, in A375 and WM1366 cell lines, respectively. Results are expressed as fold change relative to the control condition (Glucose-alone medium, 25 mM) (*** *p* < 0.0001; **** *p* < 0.0001; ANOVA followed by Tukey’s test for multiple comparisons). (D) Cathepsin B activity was evaluated using 50 μg of proteins from the lysates of each cell line toward the chromogenic substrate Z-Arg-Arg-pNA (* *p* < 0.05, *t* test was separately carried out for each cell line and their respective experimental conditions after 24 h of incubation). (E) Heatmap showing the abundance ratio [log_2_(fold change)] profile of proteins related to selected metabolic pathways in each experimental condition. Quantile-normalized log2 median protein intensity values were standardized (z-score). (F) Abundance ratio [log_2_(fold change)] of selected proteins across distinct experimental conditions in both cell lines.

To enable a deeper view of the effect of mannose on protein abundance profiles, we performed a statistical comparison (*t* test) of the quantitative proteomics data. Differentially abundant proteins under each experimental condition were observed. Although a few proteins had been found up/downregulated regardless of the experimental condition/time course, some conserved trends were observed, mainly among those involved in protein turnover pathways (Figure 4A; Tables S4 and S5). For example, the lysosomal enzyme cathepsin B was highly abundant when cells were cultured in the medium containing mannose (Figure 4A; in both 24 and 48 h for the A375 cell line and after 24 h in the WM1366 cell line). Moreover, the mitochondrial enzyme isovaleryl-CoA dehydrogenase, a protein involved in the leucine catabolic pathway, was upregulated in the A375 proteome after 24 h of incubation in the mannose medium. After 48 h, proteins involved in cell death (such as BH3-interacting domain death agonist) and redox metabolism (glutathione synthetase) were upregulated in the mannose-containing medium. Altogether, our results indicated the predominance of protein turnover pathways when cells were cultured in the medium containing mannose; therefore, we used flow cytometry to evaluate the acidic vesicular organelle (AVO) content under each experimental condition.

The AVO content significantly changed after 48 and 72 h of the incubation of melanoma cells with the medium containing mannose alone or the mixture of hexoses (Figure 4B,C), and this effect was pronounced in the A375 cell line. In addition, lysates from cells cultured with the mixture of hexoses for 24 h displayed higher activity of the lysosomal enzyme cathepsin B (Figure 4D). Indeed, most of the soluble acid hydrolases are modified with mannose-6-phosphate (M6P), and such a chemical modification is crucial for their recognition by M6P receptors in the Golgi complex, allowing their proper transport to endosomal/lysosomal system.^35^ Therefore, the accumulation of M6P in culture conditions comprised of the mixture of the hexoses may favor the transit and accumulation of M6P-modified acidic hydrolases, such as cathepsin B into the lysosomes. However, although our shotgun proteomics data revealed higher cathepsin B levels, it is not possible to rule out that the cleavage of the chromogenic substrate used in our analysis was due to other active proteases in the samples. For example, lysosomal protective protein (cathepsin A) and cathepsin Z abundances were also higher in the condition comprised of the mixture of hexoses (Table S6).

Furthermore, the formation of the autophagosome and the degradation of its cargo by lysosomal enzymes are critical steps for circumventing energetic demands in rapidly growing or nutrient-deprived cells.^36^ Our results illustrate such a scenario since the mannose-containing culture medium increased the content of acidic vesicular organelles and cathepsin B abundance/activity in melanoma cells.

In general, apart from a few proteins, culturing the cells in a medium containing mannose resulted in the downregulation of proteins related to central metabolic processes, including carbohydrate metabolism (i.e., proteins related to pentose phosphate pathway, glycolysis, and *N*-glycan metabolism (Figure 4E and Table S6). A closer look into the abundance profile of proteins related to pivotal biological processes, such as fatty acid metabolism, apoptosis, and redox (GSH) metabolism, suggested mannose-driven changes with functional implications (Figure 4F); this was the case of 3-ketoacyl-CoA thiolase, a mitochondrial enzyme that catalyzes the last step of the β-oxidation pathway, a process for breaking down fatty acids into acetyl-CoA, and whose abundance was higher in both 24 and 48 h of incubation of the A375 cell line in the medium containing the mixture of hexoses. The Bcl-2-like protein 13, which activates caspase-3 and apoptosis, displayed higher abundance after 48 h of incubation of the A375 cell line in the mannose-containing medium. In contrast, the programmed cell death protein 10 was more abundant in the WM1366 cell line after 24 h of culture in a mannose-containing medium (Figure 4F). Glutathione synthetase enzyme also displayed a higher abundance in the WM1366 cell line after 48 h of culture in the mannose medium.

These proteins represent the metabolic reprogramming that these cells have undergone to adapt to mannose as a hexose source. Although both cell lines also had glucose available in the culture medium, our results reveal how metabolism was redirected to control proliferation and cell death in a limiting nutrient condition.

### Mannose Affected N-Terminal Acetylation, the Nature of N-Termini, and the Profile of Active Proteases in Melanoma Cell Lines

**Figure 5:**
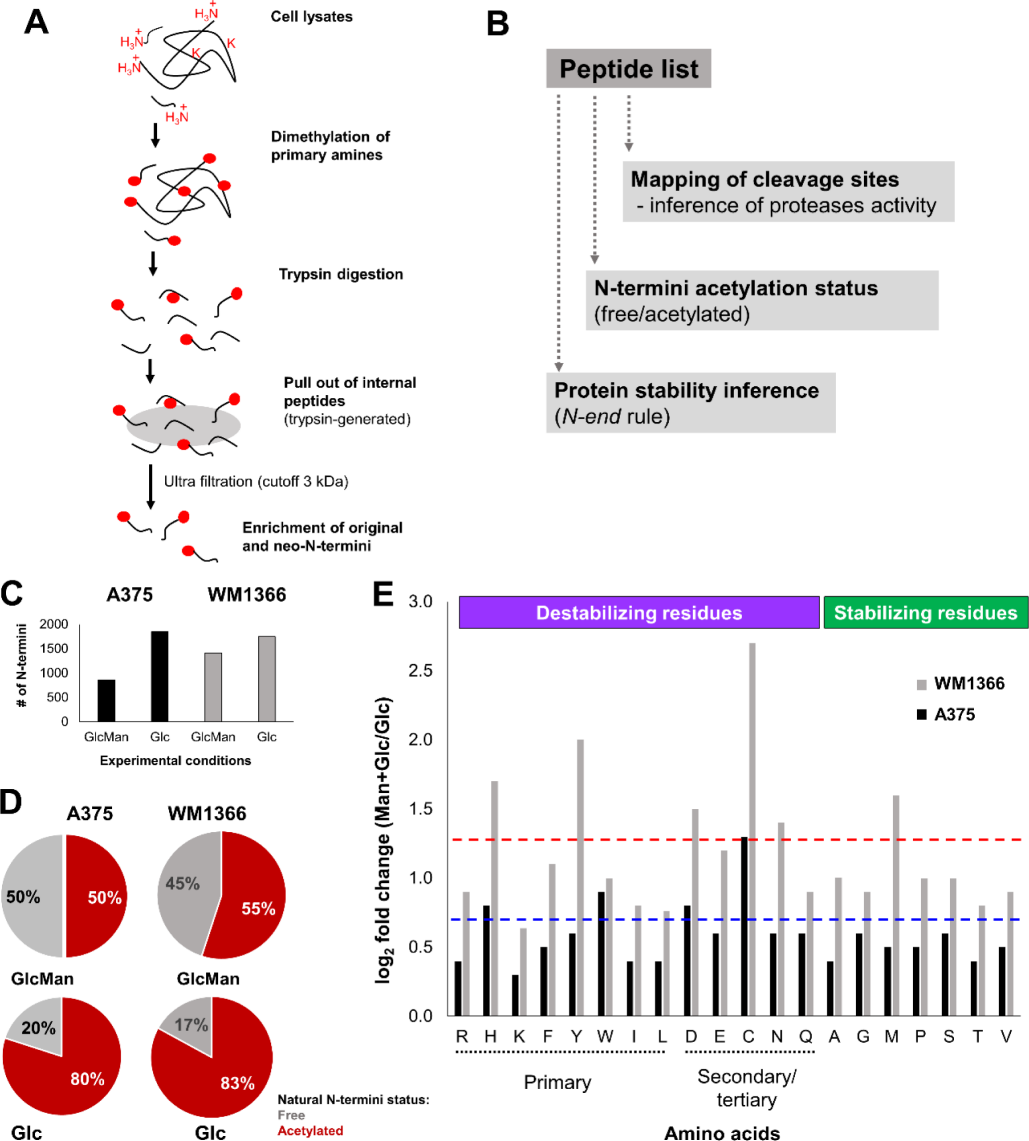
Mannose affects N-terminal acetylation and the nature of N-termini. (A) Schematic representation of the TAILS approach and (B) data analysis. Mannose affected the N-terminome of melanoma cells’ proteins quantitatively (C) and qualitatively (D and E). (D) Proportion of N-terminal acetylation in both experimental conditions. (E) The cell line that displayed higher MPI activity (WM1366 cell line) showed a higher proportion of primary and secondary destabilizing residues (N-end rule); dashed lines represent the mean of log_2_(fold change) values in both A375 (blue) and WM1366 (red) cell lines.

As we observed changes in processes related to protein turnover, we aimed to investigate the degradome of melanoma cell lysates using terminal amine isotopic labeling of substrates (TAILS), an N-terminomics analytical approach that allows the profiling of proteolytic events and the determination of natural N-termini (N-terminome) in complex biological samples^22,37^ (Figure 5A,B). The number of unique N-termini was higher when cells were cultured in the medium with glucose as the only source of hexoses, and this difference was better evidenced in the TAILS analysis of the A375 cell lysate proteins (Figure 5C; Tables S7–S10). In addition, the acetylation status, a recurrent cotranslational protein modification found in the N-termini of eukaryotic proteins,^38^ displayed similar profiles in both A375 and WM1366 when the cell lines were cultured in glucose medium but significantly changed after culturing cells in the medium with the mixture of hexoses. More specifically, the ratios of free to acetylated N-termini were similar for both cell lines at either culture condition; however, the culture medium in which mannose was added resulted in approximately 30% less acetylation in the natural N-termini of identified proteins (Figure 5D).

Since the TAILS approach allows the identification of the general pool of protein N-termini, either unprocessed (“original”) or generated by active proteases in the sample, we next evaluated the identity of the first amino acid in the whole set of identified peptides obtained by the TAILS protocol. Such an analysis showed that each cell line responded slightly differently to adding mannose to the culture medium. As the identity of the protein’s first amino acid is related to its half-live, a process known as the N-end rule,^39,40^ our results suggested that the cell line that displayed higher MPI activity (WM1366 cell line) showed a higher proportion of primary and secondary destabilizing residues when cultured in medium containing mannose (Figure 5E). This observation is in line with our results from the analysis of the WM1366 cell line proteome, suggesting that the cell with the higher activity of MPI (thus less sensitive to the mannose treatment) displayed significant alterations in its proteostasis status, a likely outcome of decreased protein stability. Moreover, several substrates identified by TAILS are involved in central metabolic pathways, such as lipid and carbohydrate metabolism, as well as protein modification and proteostasis processes, such as protein ubiquitination and SUMOylation (Figure S4). However, the functional implications of these processed substrates to the melanoma cells remain to be further evaluated.

**Figure 6:**
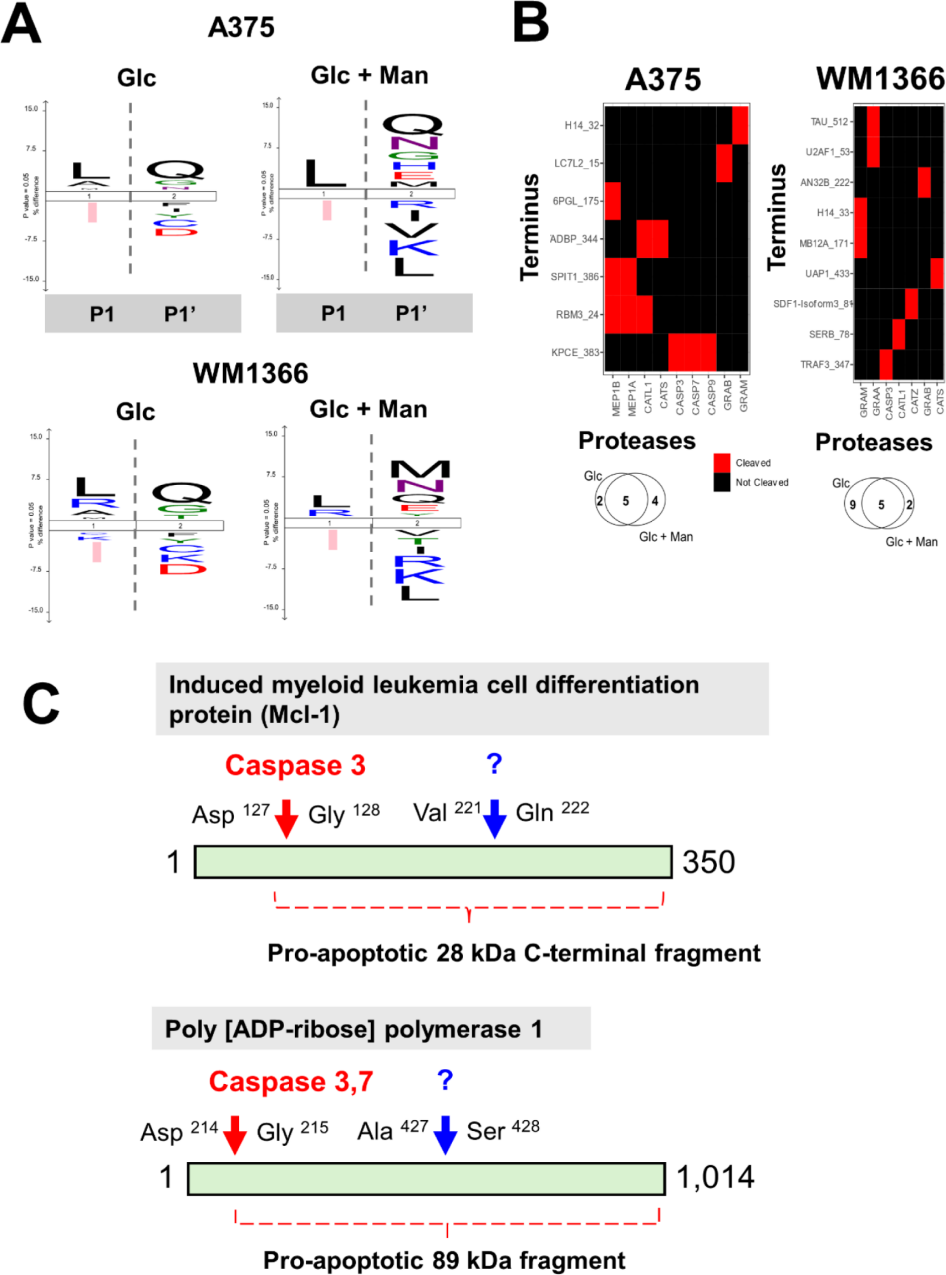
Mannose affects the degradome of active proteases in melanoma cell lines. (A) Sequence logos showing the adjusted frequency of amino acids at the scissile bond (P1 and P1’ positions) based on the mapping of the cleavage sites in the identified substrates obtained by TAILS. Vertical dashed lines denote the scissile bond. (B) Upper panel: N-terminal peptides identified using the TAILS approach matched to annotated cleavage sites, suggesting potentially active proteases in cell culture condition comprised of the mixture of hexoses (Glucose + Mannose, 12.5 mM each); lower panel: Venn diagrams showing the number of potentially active proteases identified after matching peptide sequences in the TopFIND database^30^ under the two experimental conditions used in this study. (C) N-terminomics allowed the identification of proapoptotic protein fragments derived from proteins such as Mcl-1 and PARP1. Red arrows indicate known caspase cleavage sites and blue arrows show the cleavage sites identified in this study through TAILS and whose protease responsible is unknown.

Additionally, mapping the cleavage sites in the identified substrates revealed a recurrence for Leu and Gln at P1 and P1’ positions, respectively, in both cell lysates (Figure 6A). In addition, for both cell lines, we observed a higher diversity of amino acids at the P1’ position in the cell culture condition comprised of the mixture of hexoses. Although the identification of the protease(s) responsible for generating such cleavage sites cannot be anticipated based solely on the mapping of the identity of amino acids at the scissile bond, the diversity observed mainly at the P1’ position likely reflects distinct active proteases after the addition of mannose in culture medium. Next, we submitted all the identified N-termini to a curated database containing annotated proteolytic processing events.^30^ We matched previously known cleavage sites, which differed when cells were cultured in either glucose alone or a mixture of hexoses (Figure 6B). Overall, the mapping of cleavage sites in melanoma cell lysates suggested the activity of a few proteases exclusively when cells were cultured with the mixture of hexoses, including the cysteine proteases and caspases 3, 7, and 9 only in the A375 cell line. In fact, in the protein lysate from the A375 cell line, we identified peptides spanning the region of proapoptotic fragments in induced myeloid leukemia cell differentiation protein (Mcl-1) and poly(ADP-ribose)polymerase 1 (PARP) protein (Figure 6C). Both Mcl-1 and PARP are physiological substrates of caspases 3 and 7, and their respective 28 and 89 kDa C-terminal fragments display proapoptotic properties.^41,42^

In line with these results, we found that after 72 h of incubation of the A375 cell line in the medium containing mannose alone, there was a significant increase in cell death, as observed by the percentage of cells in the sub-G1 phase of the cell cycle (Figure S5). On the other hand, no significant difference was observed for the WM1366 cell line in either of the experimental conditions. In summary, the mapping of cleavage sites obtained by the TAILS approach expanded our analyses of the effect of mannose in melanoma cells, as it allowed for the profiling of potentially active proteases whose identities could not be observed by our shotgun proteomics analysis.

Our findings suggest that the distinct activity of mannose-6-phosphate isomerase (MPI) directly correlates with the sensitivity to mannose. As the energy demand necessary for biosynthetic processes and cell proliferation is increased in the BRAF mutant A375 cell line, some key catabolic pathways were significantly affected upon mannose treatment, including the glycolytic flux as well as the induction of the apoptotic process through the activation of caspases. Conversely, although mannose had induced significant molecular changes in the proteome/degradome of the cell line with the higher MPI activity (WM1366 cell line), its effects were counterbalanced mainly by the alteration in protein stability, likely due to the activation of protein catabolic pathways to fulfill energetic demands. Thus, it is possible to infer that the low expression profile of the MPI gene led to a rewiring of metabolic pathways when cells were exposed to a culture medium containing mannose. Interestingly, the low expression profile of MPI does not seem to be exclusive to transformed cells; instead, such a pattern of expression is a signature of the skin tissue.

Furthermore, the functional status of mannose-6-phosphate isomerase caused significant downstream effects; for example, the metabolic constraints resulting from mannose administration upon melanoma cells led to the lack of pyruvate, therefore lowering the levels of its derivative metabolite, acetyl-CoA. Indeed, we observed an increased abundance of 3-ketoacetyl-CoA thiolase, a key enzyme involved in the β-oxidation of fatty acids, as a likely attempt of cells to replenish acetyl-CoA levels. Acetyl-CoA is the substrate for acetyltransferases such as *N*-acetyltransferases (which are involved in protein N-terminal acetylation) and lysine acetyltransferases. Indeed, we observed lower acetylation in the N-termini of proteins from both cells after the treatment with the mixture of hexoses. Whether such a result is directly related to lower levels of acetyl-CoA must be further investigated. Disturbances in the levels of acetyl-CoA may also affect epigenetic signaling through histone acetylation and transcriptional regulation.^43−45^ Although further studies on the downstream effectors affected by the administration of mannose are needed, the functional status of mannose-6-phosphate isomerase may prove to be a promising target when exploring adjuvant therapies for melanoma treatment.

## Conclusions

In this work, we have expanded the characterization of the molecular effects of mannose in tumoral cells including mapping some proteolytic signaling events related to cell death. Given the high heterogeneity of somatic mutations in melanoma tumors, it is not possible to anticipate that the molecular outcome of our findings would also be shared by tumors bearing distinct somatic mutations (i.e., other than the ones displayed in the cell lines used in this study) or whether nontransformed cells would respond in the same manner. Moreover, further investigations are needed to comprehensively understand the regulation of MPI gene expression in healthy and diseased conditions, mainly to underscore the molecular effectors that modulate the biosynthesis of MPI. Hence, such observations can be considered limitations of the study. However, we demonstrated that mannose, the epimer of glucose, provides opportunities to treat neoplasia, in which the MPI gene has low expression. In conclusion, although the MPI protein abundance and gene expression status are not prognostic markers, perturbation in the network caused by an exogenous monosaccharide source (i.e., mannose) significantly affects the downstream interconnected biological circuitry. Therefore, as reported in this study, the proteomic and degradation mapping of mannose downstream effects may help target specific molecular players from affected biological signaling circuits.

## Data Availability

The mass spectrometry proteomics data have been deposited to the Mass Spectrometry Interactive Virtual Environment (MassIVE, https://massive.ucsd.edu/ProteoSAFe/static/massive.jsp) with the dataset identifier. MassIVE MSV000094098.

## Supporting Information Available

Table S1: Output of the MaxQuant search engine “proteinGroups.txt” file, containing the identified proteins in both cell lines and in all experimental conditions. Table S2: Protein assigned to clusters generated by fuzzy c-means clustering (A375 cell line). Table S3: Protein assigned to clusters generated by fuzzy c-means clustering (WM1366 cell line). Table S4: Output of statistical analysis (*t* test) from the A375 cell line (Glc vs Glc + Man) showing differentially abundant proteins. Table S5: Output of statistical analysis (*t* test) from the WM1366 cell line (Glc vs Glc + Man) showing differentially abundant proteins. Table S6: Quantile-normalized log2 median protein intensity values and biological annotation (UniProt/Pathways) of proteins identified in shotgun proteomics experiment for both cell lines. Table S7: Search results from TAILS experiment from the A375 cell line after incubation in a medium containing Glucose alone or the mixture of hexoses (Glc + Man). Table S8: Search results from TAILS experiment from the WM1366 cell line after incubation in a medium containing Glucose alone or the mixture of hexoses (Glc + Man). Table S9: Annotation of all N-termini identified from the A375 cell line after incubation in a medium containing Glucose alone or the mixture of hexoses (Glc + Man). Table S10: Annotation of all N-termini identified from the WM1366 cell line after incubation in a medium containing Glucose alone or the mixture of hexoses (Glc + Man) (XLSX)
Figure S1: Expression profile of MPI gene and PMI protein across distinct tissues/skin cancer cell lines. Figure S2: Reproducibility of protein quantitation values between the three biological replicates. Figure S3: Protein content in each cell line/experimental condition. Figure S4: Biological pathways of substrates identified by TAILS analysis. Figure S5: Mannose-induced cell death evaluated by flow cytometry (PDF)
