# Correction to “Mannose-6-Phosphate Isomerase Functional Status Shapes a Rearrangement in the Proteome and Degradome of Mannose-Treated Melanoma Cells”

**DOI:** 10.1021/acs.jproteome.4c00998

**Published:** 2024-11-26

**Authors:** Nathália de Vasconcellos Racorti, Matheus Martinelli, Silvina Odete Bustos, Murilo Salardani, Maurício Frota Camacho, Uilla Barcick, Luis Roberto Fonseca Lima, Letícia Dias Lima Jedlicka, Claudia Barbosa Ladeira de Campos, Richard Hemmi Valente, Roger Chammas, André Zelanis

The funding information for the manuscript is missing in the online version of the article. Including these sources in the manuscript is required by the funding agency. The sources below need to be listed in the funding section (in addition to the information regarding the cost of the article processing charge).

Funding sources to be added:

Coordenação de Aperfeiçoamento de Pessoal de Nível Superior (CAPES)

Fundação de Amparo à Pesquisa do Estado de São Paulo (FAPESP) 2019/10855-0 2021/05087-3 2022/15421-0 2023/05715-0

