# Supplementary material for: Mannose-6-Phosphate Isomerase Functional Status Shapes a Rearrangement in the Proteome and Degradome of Mannose-Treated Melanoma Cells
Source: J Proteome Res. 2024 Oct 18;23(11):5177–92. doi: 10.1021/acs.jproteome.4c00705 (PMC11536439; doi:10.1021/acs.jproteome.4c00705)
Supplement: Supplementary file 2 — pr4c00705_si_003.pdf [file pr4c00705_si_003.pdf]

SUPPORTING INFORMATION

Mannose-6-phosphate isomerase functional status shapes a rearrangement in the proteome and degradome of mannose-treated melanoma cells

Nathália de Vasconcellos Racorti[1, †], Matheus Martinelli[1,†], Silvina Odete Bustos[2], Murilo Salardani[1], Maurício Frota Camacho[1], Uilla Barcick[1], Luis Roberto Fonseca Lima[1], Letícia Dias Lima Jedlicka[1,3], Claudia Barbosa Ladeira de Campos[4], Richard Hemmi Valente[5], Roger Chammas[2,6], André Zelanis[1,*]

[1]Functional Proteomics Laboratory, Federal University of São Paulo – UNIFESP, São José dos Campos, São Paulo, Brazil;
[2]Grupo de Oncologia Experimental - Instituto do Câncer do Estado de São Paulo – ICESP, São Paulo – SP, Brazil;
[3]Instituto de Estudos em Saúde e Biológicas, Universidade Federal do Sul e Sudeste do Pará- Unifesspa, Marabá-PA, Brazil;
[4]Laboratory of Biochemistry and Molecular and Cellular Biology of Fungi, Federal University of São Paulo – UNIFESP, São José dos Campos, São Paulo, Brazil.
[5]Laboratory of Toxinology, Center for Research, Innovation, and Surveillance in COVID-19 and Health Emergencies, FIOCRUZ, Rio de Janeiro, Brazil;
[6]Faculdade de Medicina da Universidade de São Paulo, São Paulo, Brazil.

[†]These authors contributed equally to this work

*Corresponding author:

André Zelanis
andre.zelanis@unifesp.br
Functional proteomics Laboratory
Department of Science and Technology
Federal University of São Paulo (ICT-UNIFESP)
São José dos Campos, São Paulo, Brazil
Rua Talim, 330, Room 202, 12231-280;
Tel +55 11 3385-4135 ext. 9727.

Table of contents

Content **Page**

Figure S1. Expression profile of MPI gene and PMI protein accross distinct tissues/skin cancer cell lines.....S3

Figure S2. Reproducibility of protein quantitation values between the three biological replicates.....S4

Figure S3. Protein content in each cell line/expermental condition.....S5

Figure S4. Biological pathways of substrates identified by TAILS analysis.....S6

Figure S5. Mannose-induced cell death evaluated by flow cytometry.....S7

A

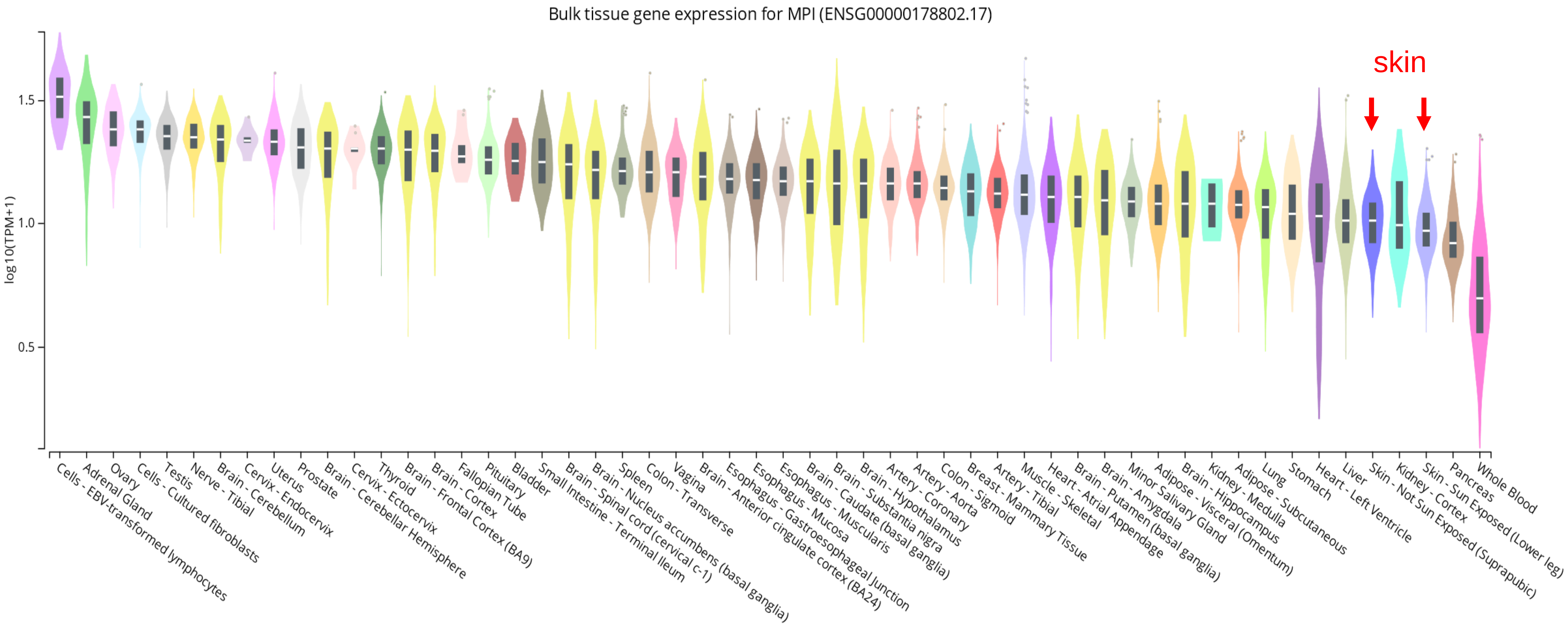

Bulk tissue gene expression for MPI (ENSG00000178802.17)
log10(TPM+1)
1.5
1.0
0.5
skin
Cells - EBV-transformed lymphocytes
Adrenal Gland
Ovary
Cells - Cultured fibroblasts
Testis
Nerve - Tibial
Brain - Cerebellum
Cervix - Endocervix
Uterus
Prostate
Brain - Cerebellar Hemisphere
Cervix - Ectocervix
Thyroid
Brain - Frontal Cortex (BA9)
Brain - Cortex
Fallopian Tube
Pituitary
Bladder
Small Intestine - Terminal Ileum
Brain - Spinal cord (cervical c-1)
Brain - Nucleus accumbens (basal ganglia)
Spleen
Colon - Transverse
Vagina
Brain - Anterior cingulate cortex (BA24)
Esophagus - Gastroesophageal Junction
Esophagus - Mucosa
Esophagus - Muscularis
Brain - Caudate (basal ganglia)
Brain - Substantia nigra
Brain - Hypothalamus
Artery - Coronary
Artery - Aorta
Colon - Sigmoid
Breast - Mammary Tissue
Artery - Tibial
Muscle - Skeletal
Heart - Atrial Appendage
Brain - Putamen (basal ganglia)
Brain - Amygdala
Minor Salivary Gland
Adipose - Visceral (Omentum)
Brain - Hippocampus
Kidney - Medulla
Adipose - Subcutaneous
Lung
Stomach
Heart - Left Ventricle
Liver
Skin - Not Sun Exposed (Suprapubic)
Kidney - Cortex
Skin - Sun Exposed (Lower leg)
Pancreas
Whole Blood

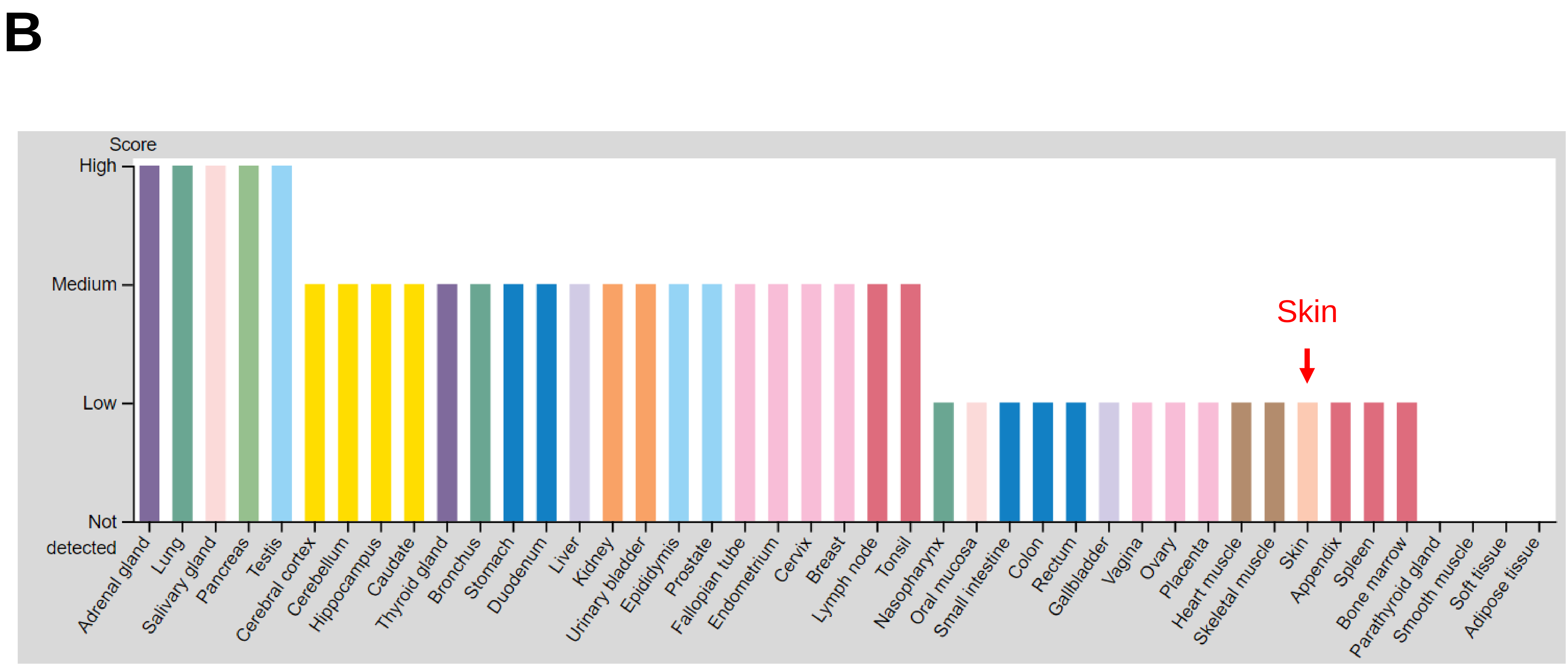
B
Score
High
Medium
Low
Not detected
Skin
Adrenal gland
Lung
Salivary gland
Pancreas
Testis
Cerebral cortex
Cerebellum
Hippocampus
Caudate
Thyroid gland
Bronchus
Stomach
Duodenum
Liver
Kidney
Urinary bladder
Epididymis
Prostate
Fallopian tube
Endometrium
Cervix
Breast
Lymph node
Tonsil
Nasopharynx
Oral mucosa
Small intestine
Colon
Rectum
Gallbladder
Vagina
Ovary
Placenta
Heart muscle
Skeletal muscle
Skin
Appendix
Spleen
Bone marrow
Parathyroid gland
Smooth muscle
Soft tissue
Adipose tissue

C

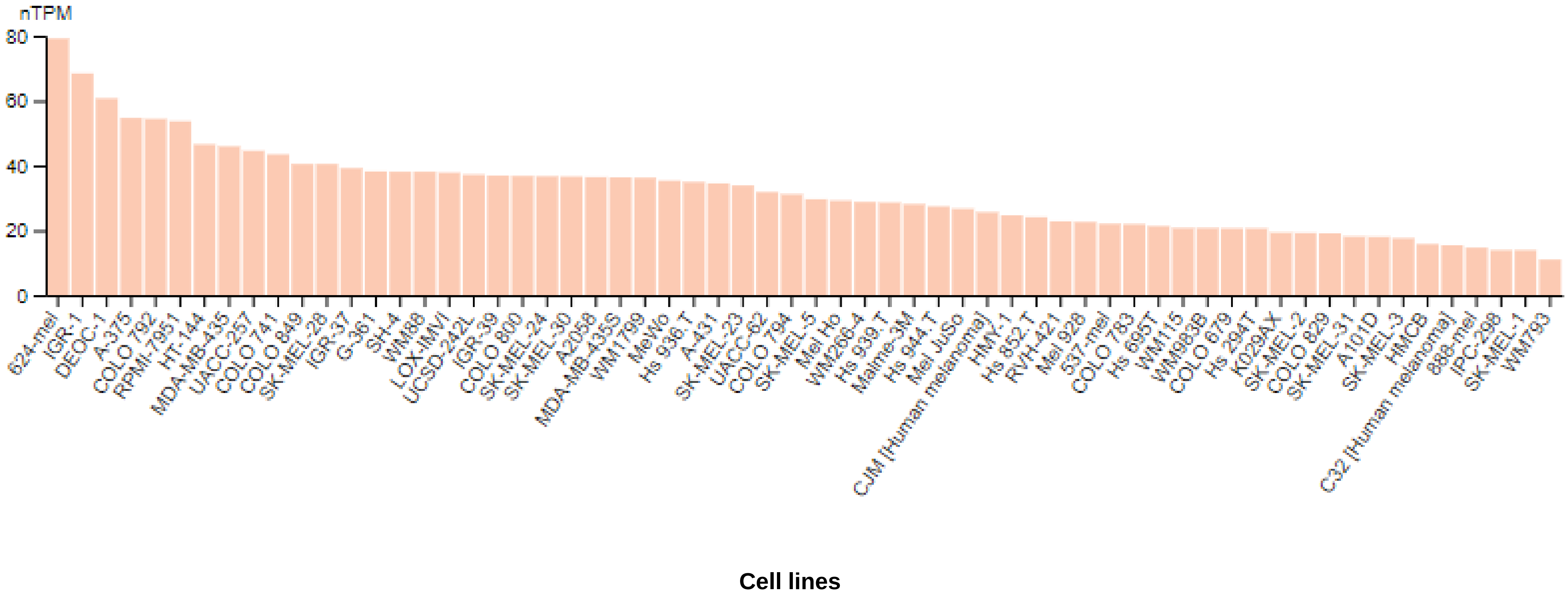

Figure S1: Expression profile of MPI gene and PMI protein. (A) Expression profiles of MPI gene across distinct tissues. Data were retrieved from Genotype-Tissue Expression (GTEx), a public repository on tissue-specific gene expression and regulation (https://www.gtexportal.org/home/gene/MPI). **(B)** Expression profiles of PMI protein across distinct tissues. Data were retrieved from the Human Protein Atlas (https://www.proteinatlas.org/ENSG00000178802-MPI/tissue). **(C)** Expression profiles of MPI gene (Transcripts per milion – TPM) across distinct skin cancer cell lines. Data were retrieved from the Human Protein Atlas (https://www.proteinatlas.org/ENSG00000178802-MPI/cell+line).

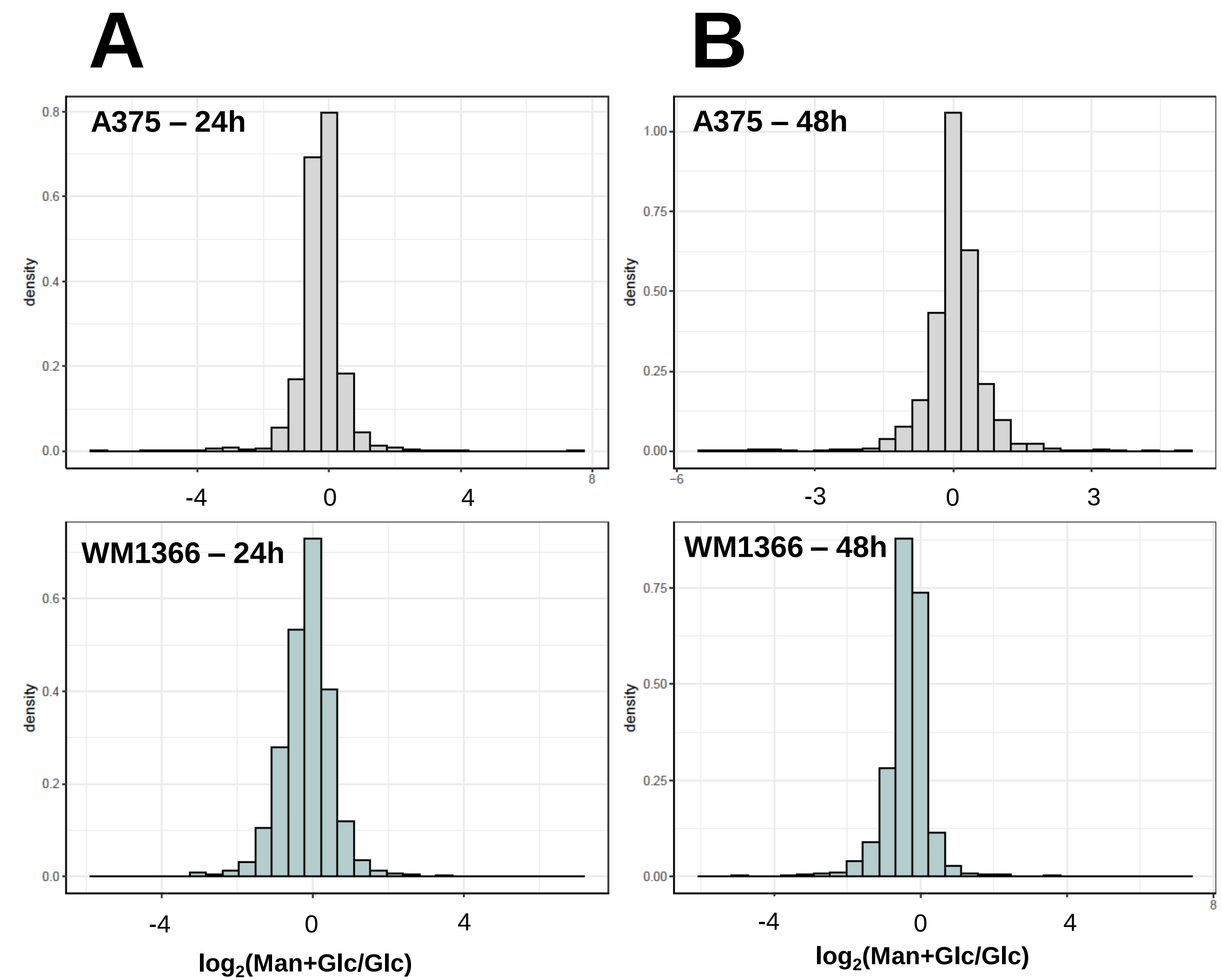

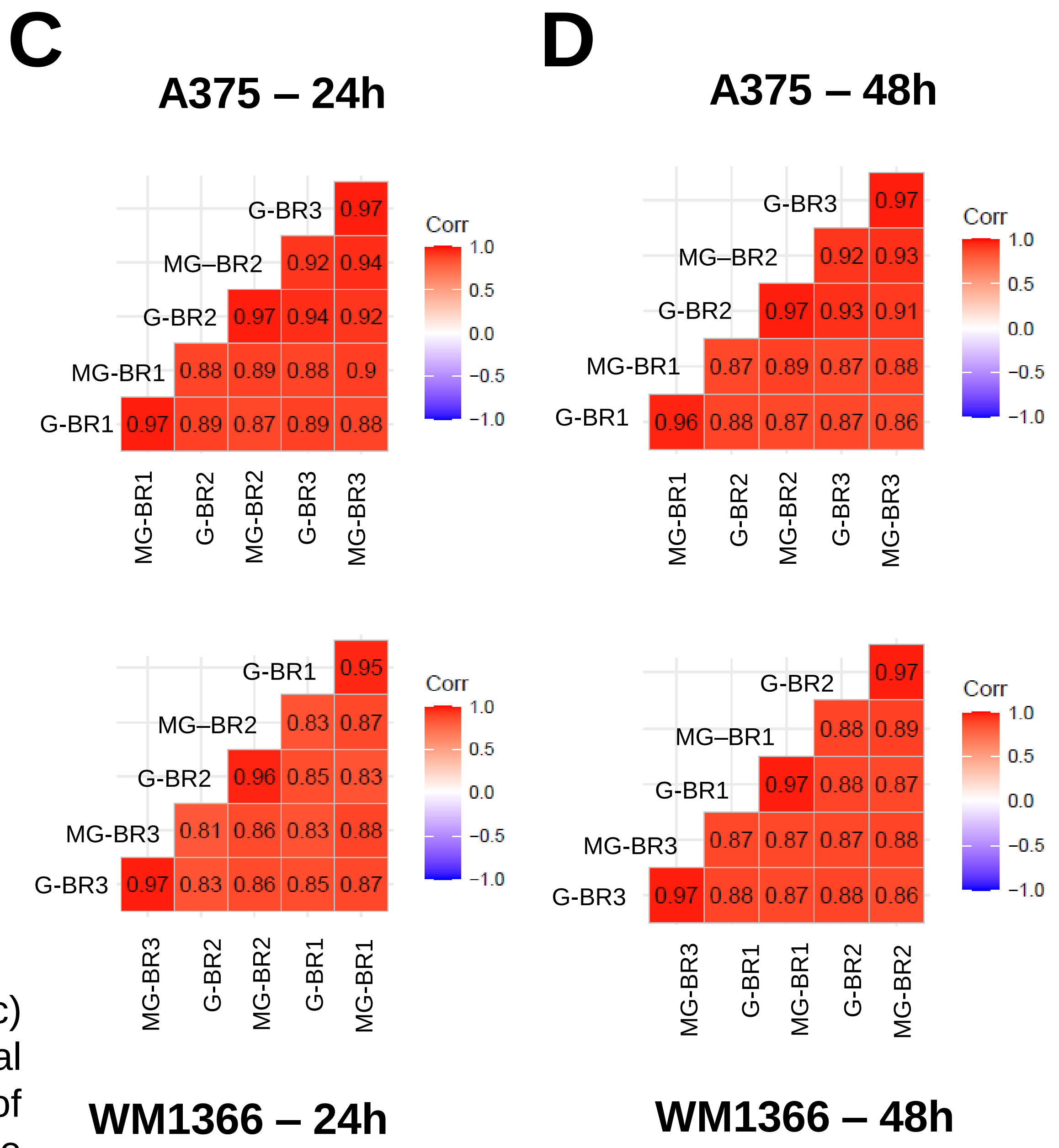

Figure S2: (A and B) Distribution of ratio values (log2(Man+Glc/Glc) for the dimethyl labeling experiment, for each cell line/experimental condition**. (C)** and **(D)** Correlation plots, showing the reproducibility of protein quantitation values (log2 (intensity L or H)) between the three biological replicates for each cell line and their corresponding Pearson correlation coefficients.

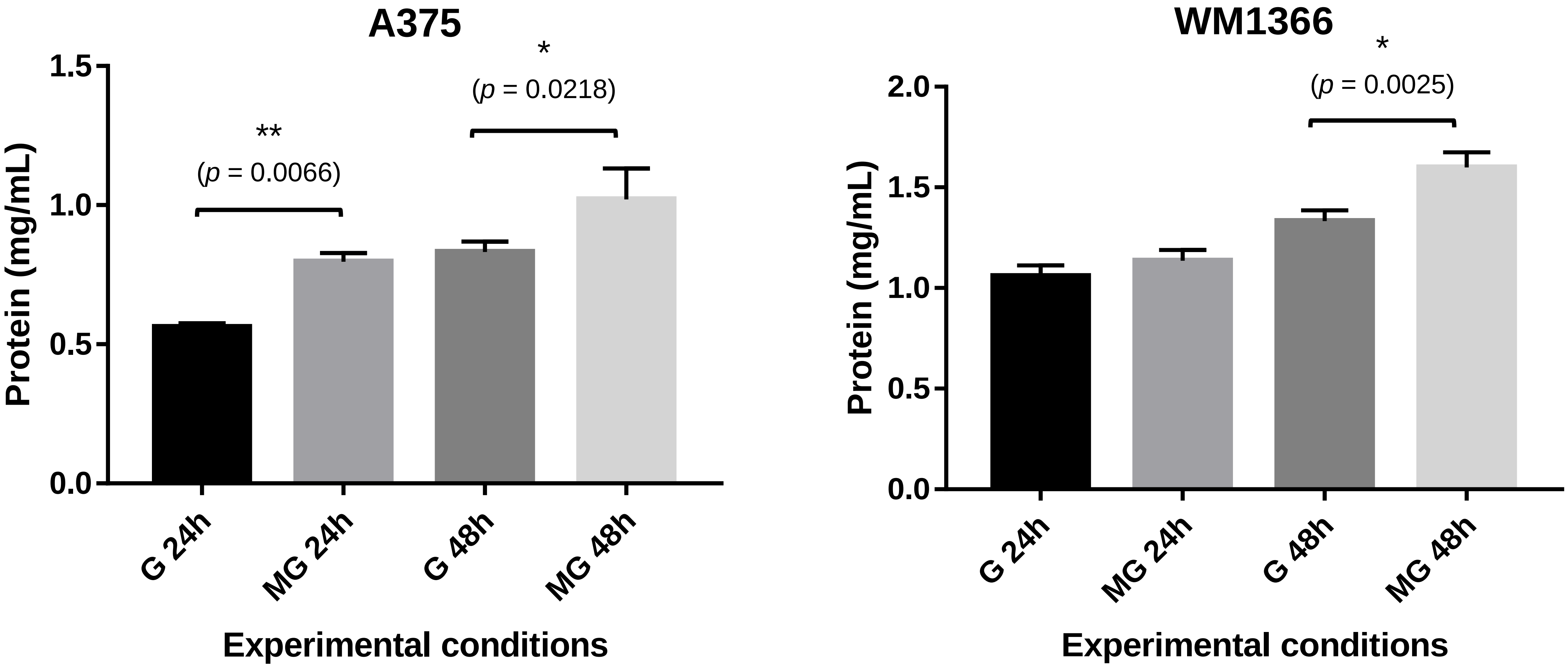

Figure S3: Protein content (measured by Bradford method) in each cell line/expermental condition. Statistical analysis (t-test) between each time point (24h or 48h) was performed in GraphPad Prism 9.

G = Glucose (25 mM)
MG = Glucose + Mannose (25 mM final)

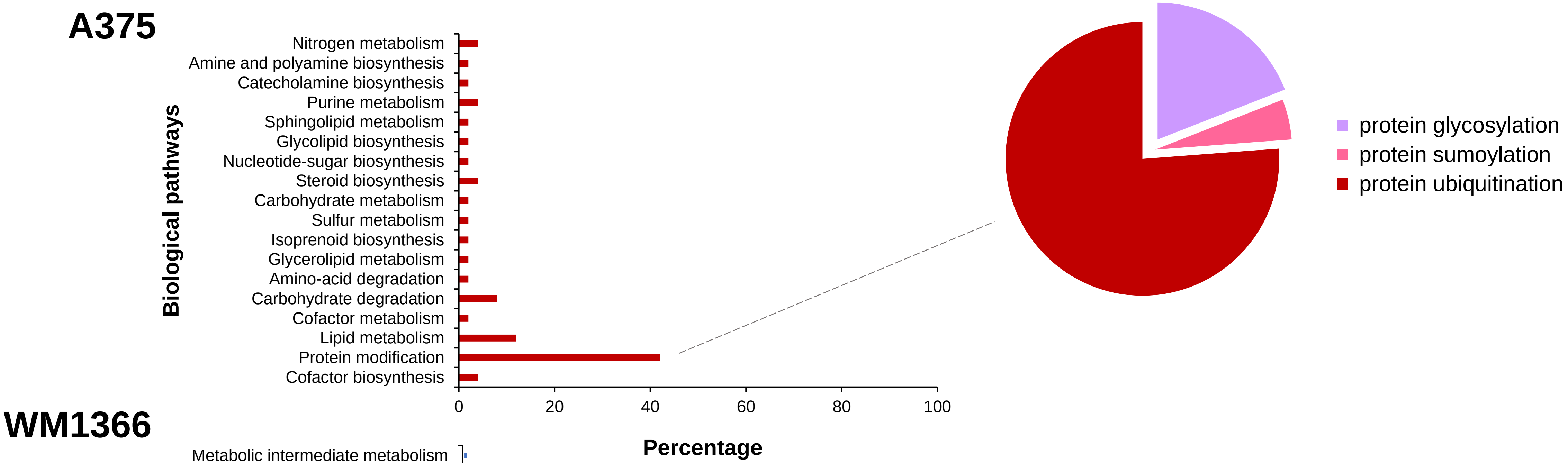

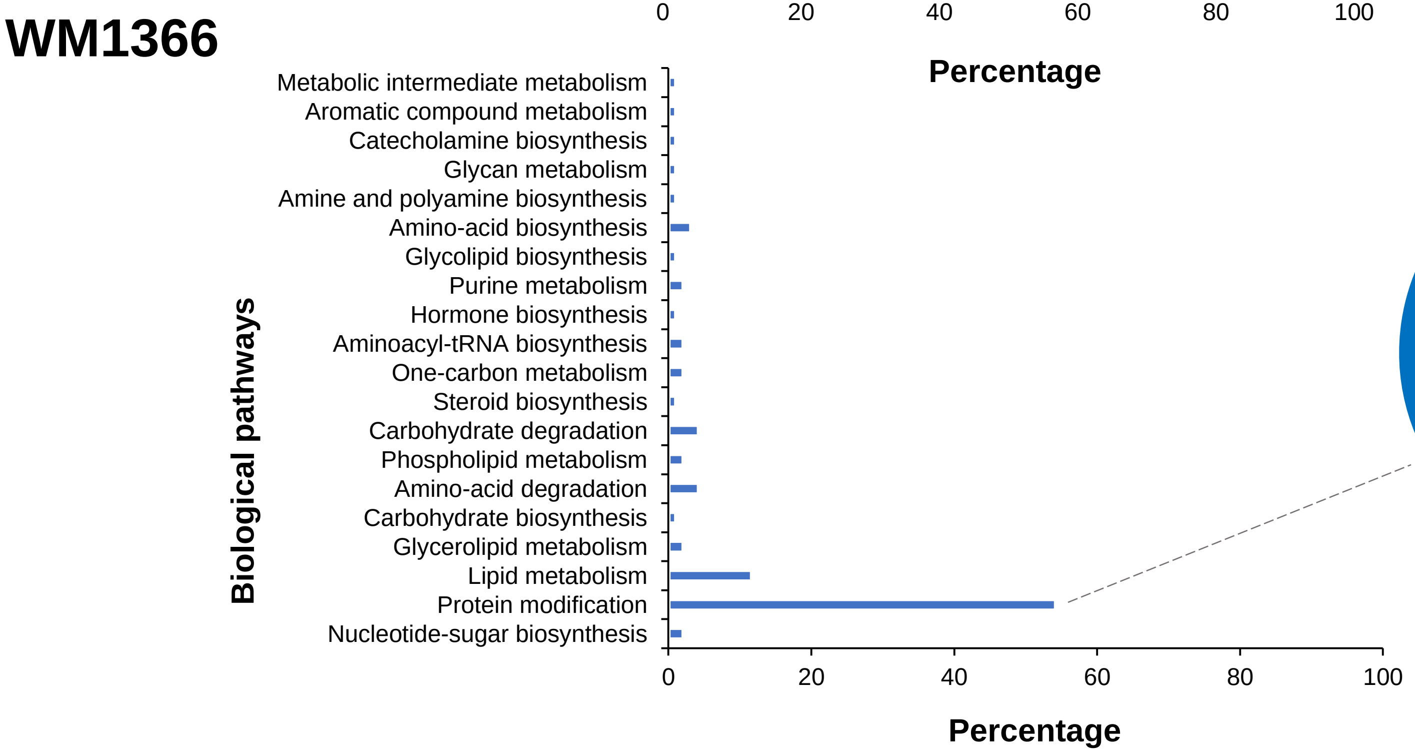

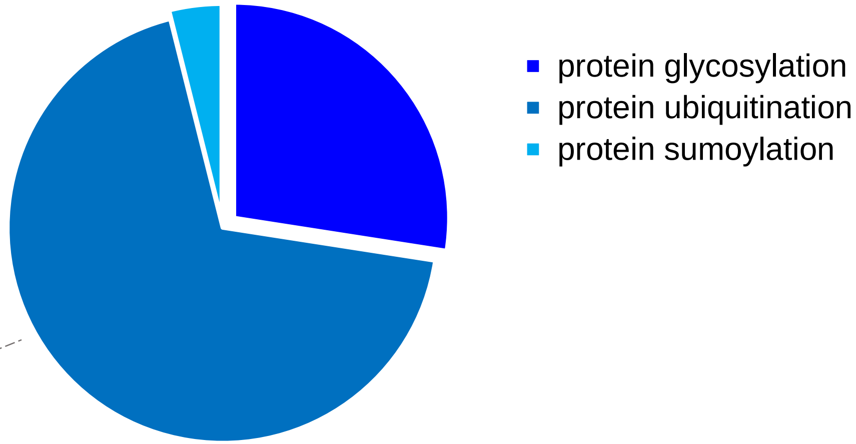

Figure S4: Biological pathways of substrates identified by TAILS analysis in both cell lysates under culturing condition comprised by the mixture of hexoses (glucose + mannose 25 mM, final).

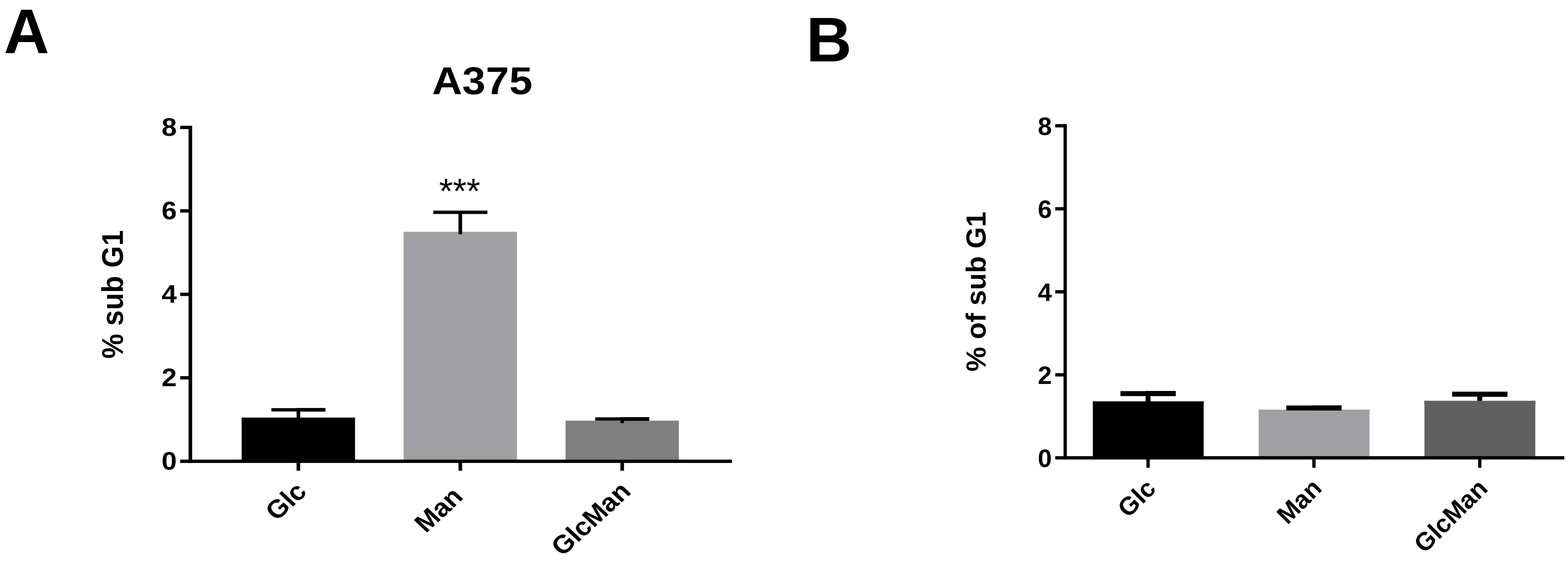

Figure S5: Mannose-induced cell death was evaluated by flow cytometry (as the percentage of cells in sub G1 phase of the cell cycle) after culturing A375 (**A**) or WM1366 (**B**) cells for 72h in media with glucose, mannose, or glucose + mannose (*** $p < 0.005$; ANOVA followed by Tukey test).
